# Influence of intergenerational support on the mental health of older people in China

**DOI:** 10.1371/journal.pone.0299986

**Published:** 2024-04-18

**Authors:** Zicheng Jiang, Huan Liu, Jing Deng, Yizhong Ye, Dexun Li

**Affiliations:** 1 School of Hospital Economics and Management, Anhui University of Chinese Medicine, Hefei, China; 2 Key Laboratory of Data Science and Innovative Development of Chinese Medicine in Anhui Province Philosophy and Social, Hefei, China; International Institute of Health Management Research - New Delhi, INDIA

## Abstract

Today, population aging is the main trend of population development. Home-based care is mainly adopted in Chinese society, and scholars have paid ample attention to the effect of intergenerational support on the mental health of older people. However, research conclusions differ. This study uses data from the 2018 China Health and Pension Tracking Survey (CHARLS), which we analyzed with STATA software to construct least squares regression and two-stage least squares regression models. The regression model included 6,647 respondents to investigate the mental health status of older people based on depression status. Intergenerational support was defined as economic support, emotional support, and daily care provided by the children of older people. We studied the impact of three aspects of intergenerational support on the mental health of the elderly. We performed a robustness test using the variable replacement and propensity score matching methods, and analyzed age, gender, and urban-rural heterogeneity. The results showed that economic support had no significant impact on the mental health of older people, while emotional support and daily care had a positive effect. The heterogeneity results indicated that the relationship between intergenerational support and mental health of older people differed significantly based on age, gender, and urban and rural areas. Therefore, children should raise their awareness of supporting their parents, pay attention to their parents’ mental health, and provide emotional support and daily care. Furthermore, community work improves family relations, creates a good social environment, and encourages young people to respect and be filial to older people. The government should improve the medical security system and old-age service system, and provide policy support to help the mental health of older people.

## Introduction

Today, population aging is the main trend of population development. According to the World population outlook: 2019 revision, by 2050, one in six people worldwide will be aged 65 years or older (16%), compared with 11 (9%) in 2019 [1]. China is one of the fastest aging countries [2]. By the end of 2021, the national population aged 60+ years totaled 260 million people, or 18.9% of the total population [3]. With the accelerating process of the aging population, the health problems of the older people comprising this population in China are receiving increasing attention. Mental health is an important aspect of healthy aging among the older population. According to global health estimates in 2019, approximately 27.2% of suicide deaths occur globally among people aged 60+ years, and around 14% of the same group experience mental disorders. The World Report on Aging and Health released by the World Health Organization in 2015 defines healthy aging as “the process of developing and maintaining functional capabilities that promote the well-being of the elderly.” The report emphasizes functional capabilities as an important consideration for healthy aging, rather than merely focusing on the absence of disease [4]. Furthermore, the mental health status of older people directly affects their participation in social activities and physical health levels, and the rupture of family and social relations caused by psychological problems seriously impacts their quality of life [5].

Maslow’s hierarchy of needs theory includes physiological needs and the needs for security, belonging and love, respect, and self-realization [6]. Here, examining the intergenerational support and mental health of older people reflects Maslow’s ownership and need for love in the hierarchy. Following Maslow, intergenerational support starts from the internal needs of older people and helps them form a healthy psychological state through satisfying needs at different levels of the hierarchy. For example, intergenerational economic support can satisfy older people’s physiological needs. Similarly, daily care and emotional support can alleviate the loneliness they experience and address their safety and social needs. In addition, when elderly people provide economic and material support such as intergenerational care to their children, they satisfy their needs for respect and self-actualization.

Filial piety is a fundamental virtue in China, which embodies a traditional Confucian culture. Thus, based on Confucianism and other traditional cultural values in China, it is expected that children express filial piety to their parents [7]. Filial piety refers to “the obedience and service of children to their parents, including respect, living with their parents (or maintaining intimate relationships in cases where they cannot live together), and taking care of their parents whether they are healthy or sick” [8]. The concept is a key factor affecting the sustainable stability of intergenerational support. Children with a stronger sense of filial piety will shoulder more “support” obligations and provide their parents with more economic and emotional support, and daily care. Older people also expect support from their adult children, especially in rural areas, where expectations for their children are greater than those of their spouses [9, 10].

Depression is the most important determinant of mental health status. People with depressive tendencies or depression exhibit a sub-healthy state of mind and may have serious psychological problems [5]. Depressive symptoms and social support are largely coherent in later life, in that lower social support predicts higher depressive symptoms [11]. Currently, the older-age care model in Chinese society is based on home-based care [12]. In the home-based care model, the majority of older people still expect their children’s filial piety and their timely help [8]. Thus, children’s intergenerational support has become the main component of social support for older people. These children also bear the responsibilities of providing economic and emotional support, and daily care, which significantly impacts older people’s mental health [13].

### Literature review and theoretical assumptions

Social exchange theory forms the theoretical foundation of this study. Developed by American sociologist Horseman, the theory is based on the “rational economic man hypothesis” in classical economics, “reciprocity principle” in anthropology, and behaviorist psychology [14]. Social exchange theory was used to explain the relationship between mental health and intergenerational exchange partly based on the economic concepts of cost and benefit [15]. The theory advances reciprocity and balance as the core driving forces of the sustainable continuation of intergenerational exchange relationships. Reciprocity means that both parties in the exchange benefit from the exchange relationship, regardless of whether the benefits are past or present, or long or short term. Following social exchange theory, social support is delineated as receiving and providing support according to different directions of this support [16]. Since the latter half of the 20th century, social exchange theory has been applied to the study of intergenerational support between the elderly and adult children [16]. The reciprocity of intergenerational support and two aspects of receiving and providing support may impact older people’s mental health [17, 18]. For example, older people may provide intergenerational support to their children in return for receiving economic and emotional support, and daily care in their later years [19]. This support from their children increases older people’s happiness and improves their mental health [20].

Some scholars believe that children’s economic support can significantly improve the mental health of older people in rural areas [21, 22]. Thus, the life satisfaction of older people may be enhanced through this support, which helps to meet their basic living and medical needs and can alleviate cognitive decline [23–26]. While economic support is material, older people also need their children’s emotional support. Children’s emotional support is closely related to older people’s health, and can reportedly improve the recovery of their physical functions [27], more so than economic support and living care [28]. Finally, the daily care children provide improves older people’s quality of life, their level of depression, and life satisfaction [29]. Importantly, a lack of daily care can reduce older people’s welfare and may negatively impact their psychological and mental health [30].

When intergenerational support lacks reciprocity or is imbalanced, insufficient benefits can lead to unmet needs and resentment. However, excessive benefits can lead to guilt or dependence. All this can increase depressive symptoms among older people [31]. Scholars holding this view believe that too much intergenerational support may reduce older people’s sense of self-efficacy; damage their self-esteem; and produce a sense of failure, guilt, and incompetence [32, 33], increasing their depression [34]. In addition, an intergenerational gap means children may be unable to give enough understanding and support to older people, which also seriously affects their mental health [35]. However, others found no significant correlation between economic support and daily care provided by children and older people’s life satisfaction, which improved through economic independence [36]. Note though that survey data at the societal level in China showed that children’s economic support had no major influence on their older parents’ life satisfaction, health, and level of depression [37, 38]. The effects of daily care were also not considerably positive. In fact, daily care accelerated the decline of cognitive function. Thus, we conclude that intergenerational support will bring about different changes depending on the needs of older people [22]. Economic support with more children also increases health risks for older adults [38]. Finally, some have found no statistically significant relationship between children’s emotional support and older adults’ self-rated health status [39].

Furthermore, in terms of gender differences, emotional support had a greater impact on the subjective health of older female adults [40]. Children’s emotional support also helps to maintain and improve older adults’ psychological status [41, 42]. However, there are significant differences in the depression status of older people in urban and rural areas in China. This may be because the economic conditions in rural areas are worse than those in urban regions. As such, the modes of intergenerational support differ between urban and rural areas, resulting in different impacts on mental health [43].

A manifestation of cultural differences between China and the West is that Chinese society follows a feedback model where intergenerational support flows in both directions between two generations [44]. From this perspective, Chinese sociologist Xiaotong Fei believes that “raising children to prevent aging” is the foundation of the feedback model, that is, the parent-child relationship is the most basic social relationship that includes two primary tasks: first, raising (parents are responsible for raising their children), and second, supporting (children are obligated to support their parents). As a constituent unit of society, the family develops and grows with continuous intergenerational support and assistance. Older people expect their children to support them and repay their upbringing when they reach adulthood. Therefore, older people who receive more support from their children will experience less depression, loneliness, and malnutrition [45]. The feedback model of intergenerational support in Chinese society emphasizes the reciprocity of support, which can strengthen family ties and provide a social support system for older people. This support system, which includes economic assistance and emotional connection, plays a crucial role in maintaining older people’s mental health and reducing the likelihood of depressive symptoms [46, 47].

Theoretically, the social exchange and feedback theories reflect the relationship and mechanism between intergenerational relationships and health, providing us with a theoretical perspective and insights into how to understand these aspects. Intergenerational support from children provides financial help and spiritual comfort, and affects their older parents’ subjective well-being and loneliness. Mental health is affected through this mental process.

**Fig 1:**
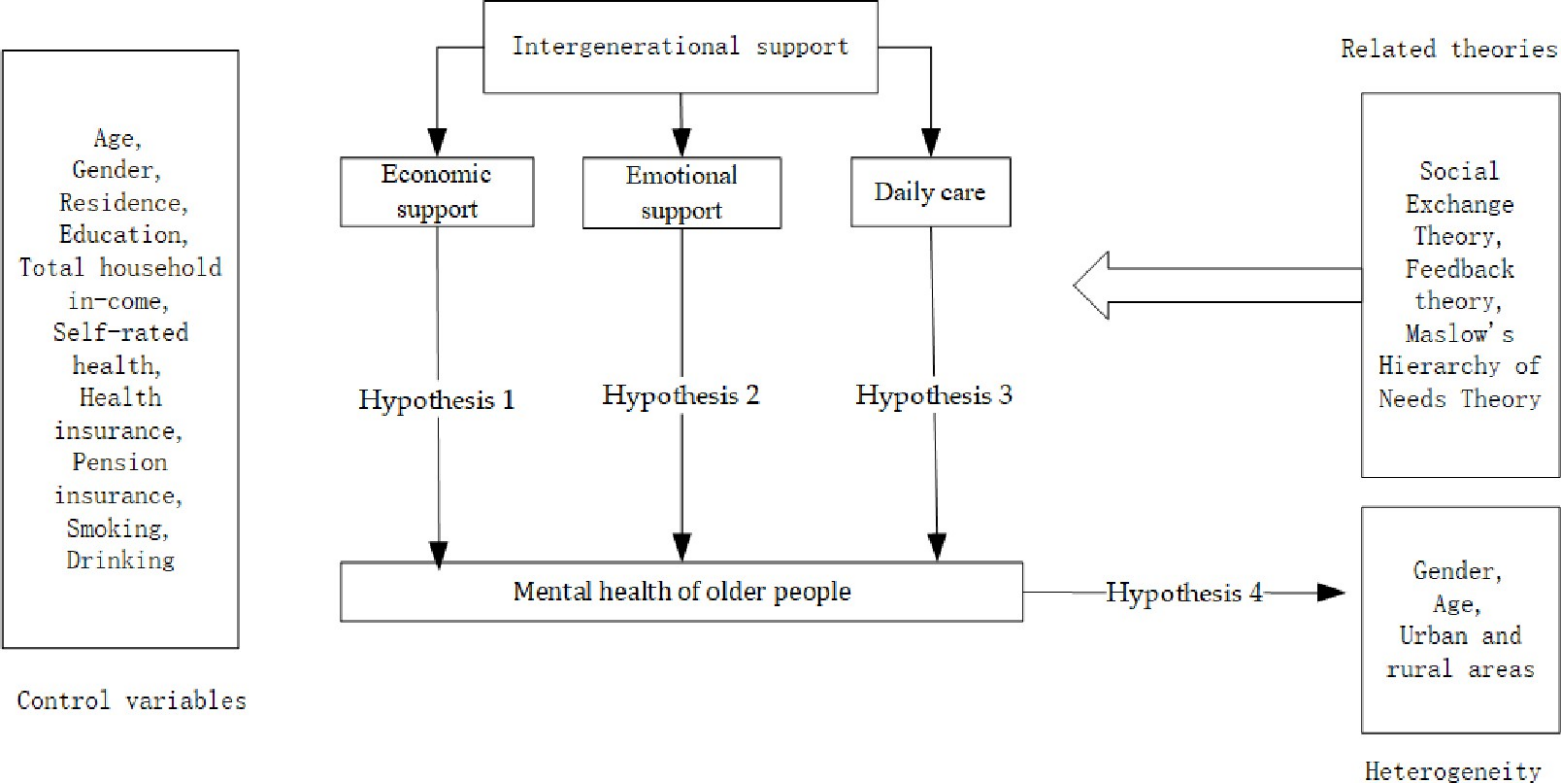
Conceptual framework of intergenerational support and mental health.

However, empirically, there is no consensus in the academic community on the impact of intergenerational support on the mental health of older people, and various conclusions have been derived depending on the theoretical perspective applied. In addition, differences in the cultural background and welfare systems between China and the West, as well as intergenerational support in terms of gender, age, and place of residence, also influence mental health in different ways. Thus, based on the above discussion, whether the different types of intergenerational support positively or negatively affect older people’s mental health should be considered in the context of Chinese culture considering their actual needs. Therefore, this study proposes the following four research hypotheses and the conceptual framework of intergenerational support and mental health (see Fig 1):

- Hypothesis 1: Intergenerational economic support has no significant impact on the mental health of Chinese older people.
- Hypothesis 2: Emotional support has a positive impact on the mental health of Chinese older people.
- Hypothesis 3: Daily care has a positive impact on the mental health of Chinese older people.
- Hypothesis 4: Intergenerational support has significant differences in terms of age, gender, and place of residence in the mental health of Chinese older people.

Through the theoretical and literature analysis, we identified three research gaps. First, previous studies on intergenerational support were mostly regional, and research on older people’s mental health and intergenerational support was scant at the national level. In addition, previous work used long-term data, which did not necessarily focus on intergenerational support. This cannot accurately reflect the current impact of intergenerational support on the mental health of the older population in China. Second, regarding research methods, an OLS regression or multivariate regression analysis was mostly used in previous studies. Furthermore, causal identification was lacking in general, because these studies were mostly descriptive or correlational [19, 20, 28]. Moreover, few studies focused on differences on the effects of gender, age, residence, and so on. However, the differences in the roles played by men and women in Chinese families, differences in the physical and mental conditions of older people of different age groups, and imbalanced urban-rural differences in developing countries result in different resource conditions and health needs. Thus, investigating the heterogeneity of intergenerational support will help to better understand healthy aging in family settings. Therefore, based on the survey data of China in 2018, this study considers the impact of intergenerational support on older people’s mental health from three aspects: economic support, emotional support, and daily care. It overcomes the problem of two-way causality and sample selection bias through the OLS model and 2SLS model, as well as the substitution variable method and propensity score matching method (PSM). This ensures more robust results. Finally, differences in gender, age, and place of residence were analyzed, and the structural differences of intergenerational support on the mental health of older people were systematically evaluated.

## Materials and methods

### Data sources

Data for this study were obtained from the China Health and Retirement Longitudinal Study (CHARLS). CHARLS contains high-quality microdata for households and individuals aged 45 years and over in China. It is used to analyze the ageing of the population and promote interdisciplinary research on ageing. The CHARLS National Baseline Survey was conducted in 2011 and covered 150 county units and 450 village units, approximately 17,000 people in 10,000 households. The questionnaire design of CHARLS referred to international works, and the data quality is generally recognized internationally as well. To ensure unbiased and representative sample selection, CHARLS sampling is completed through four stages: sampling at the county (district), village (residence), household, and individual levels. Specifically, for sampling at the county (district) and village (residential) levels, CHARLS adopts probabilities sampling proportional to population size sampling [48]. Strict quality control is carried out at all stages of the data collection process (e.g., interviewer training and on-site visits, etc.), including field organization management, field visit quality control, and real-time coding of important information, to ensure the standardization of visits and accuracy of data [48]. Chen [49] outlines more goals and methods of CHARLS. All data collected by the project were maintained at the Peking University Institute for Social Sciences Research and published on the CHARLS project (http://charls.pku.edu.cn) website. One can access the database after approval of registration. CHARLS was approved by the Biomedical Ethics Review Board, Peking University, China (IRB00001052-11,015). Finally, all participants provided their written informed consent.

The data for the purposes of this study were selected from the National Baseline Survey of CHARLS 2018. According to the Chinese Law on the Protection of the Rights and Interests of the Elderly, people over the age of 60 years are designated as elderly (referred to as older people in this paper). The target population for this study was older people aged 60+ years. Through cleaning the relevant data and removing the missing and unreasonable samples of key variables, our final valid sample included 6,647 people.

### Variable selection

#### Dependent variable

The dependent variable for this study is mental health. Depression, a common mental disease, is the main cause of suicide [50]. As such, psychologists measure the mental health of middle-aged and older people by assessing depression. The 10-item version of the Center for Epidemiological Studies Depression Scale(CESD-10) is widely used to assess depressive symptoms to measure the mental health of older people, and has been proven to have good reliability and validity [51]. The response options in the CESD-10 for each item are as follows: “little or no, < 1 day”; “Not too much, 1–2 days”; “Sometimes or half the time, 3–4 days”; and “most of the time, 5–7 days.” These are rated as 0, 1, 2, and 3, respectively. In the questionnaire, if a respondent failed or refused to answer several of the questions, it meant that the respondent had not experienced that situation and thus, a value of 0 was uniformly assigned. Note that the items “I am full of hope for the future” and “I am very happy” were positive and were reversely coded. A higher score indicated a higher depressive mood. The score ranged from 0 to 30.

#### Independent variables

The explanatory variable in this study is intergenerational support [13, 52, 53]. In this study, intergenerational support is measured from three aspects: economic support, emotional support, and daily care. Economic support refers to whether parents received food and cash support from all their children in the past year. It is coded as 1, not 0. Emotional support refers to how long it takes to see children when parents and children do not live together. We assigned a value of 1 for once every six months or more, and 0 otherwise. Daily care was assigned a value of 1, not 0, depending on whether or not the parent resides with the child.

#### Control variables

The control variables include socio-demographic, family, social, and economic characteristics [5, 54]. Table 1 defines the variables and their type.

**Table 1:** Variable definitions and types.

Variable name	Variable definition
*Independent variables*	
Economic support	Amount of food and cash support parents received from all children in the past year: No economic support received = 0 Received economic support = 1 Intergenerational economic support refers to “upward” intergenerational economic support.
Emotional support	Item: “How often do you see [XChildName[i]] when you and [XChildName[i]] are not living together?” Other = 0 Biannual and above = 1. Emotional support refers to “upward” emotional support.
Daily care	Whether there are children living in the same family: None = 0, Yes = 1
*Dependent variable*	
Depressive condition	Little or no, < 1 day = 0 (including not knowing, refusing to answer) Not too much, 1–2 days = 1 Sometimes or half the time, 3–4 days = 2 Most of the time, 5–7 days = 3
*Control variables*	
Age	The year of the questionnaire minus the year of birth gives the age: 60–70 years = 1 70–80 = 2 80+ = 3
Gender	Female = 0 Male = 1
Place of residence	Rural = 0 Urban and rural = 1
Marital status	No spouse (including divorced, widowed, and never married) = 0 Spouse (including married living with a spouse, married but not temporarily living with a spouse, separated) = 1
Education of older people	Illiterate = 1 (i.e., no education) Primary school and below (including unfinished primary school, private school graduation, primary school graduation) = 2 Junior high school or high school (including junior high school graduation, high school graduation, technical secondary school including secondary normal, vocational high school graduation) = 3 College and above = 4
Total household income	Adding up all property income of the family; Logarithmic processing
Self-rated health	Good or very good = 1 General, bad, or very bad = 0
Health insurance	Based on the item: “Are you currently enrolled in 10 health insurance policies?” Not participating in any coverage type = 0 Participate in either = 1
Pension insurance	According to the item: “Are you receiving or expecting to receive or paying the following residents’ pension insurance in the future, including urban and rural residents’ pension insurance, new rural pension insurance, and urban residents’ pension insurance?” Not participating = 0 Participating = 1
Smokes	No/Never = 0 Yes/Has = 1
Drinks wine/alcohol	No/Never = 0 Yes/Has = 1
*Instrumental variable*	
Does the child work	No/Never = 0, Yes/Has = 1

#### Instrumental variables

Other confounding factors beyond intergenerational support may influence the results of the model. Thus, to avoid reverse causality [55], we consider whether children work or not as a tool variable. Whether children are employed or not can reflect their economic status on one hand, and on the other, their work can affect the level of emotional support and time of daily care provided. However, children’s work has no direct connection with the mental health of older people, and therefore, can be used as a instrumental variable.

### Model setting

As the dependent depression scores are continuous variable, the OLS model selected is applicable for this study. The model settings are as follows:

$$H_{i}=\beta_{0}+\beta_{1}{\text{involvement}}_{i}+\beta_{2}financial+\beta_{3}emotional+\beta_{4}routine+\lambda X_{k}+\varepsilon \;(k=1,2\ldots ,n)$$ (1)

To avoid the problem of endogeneity, we use a two-stage (2SLS) instrumental variable model:

$$Stage\;1:Y_{j}=\alpha_{1}Ζ1_{i}+\alpha_{2}Ζ2_{i}+\gamma {Χ}_{k}+\theta_{i}\;(k=1,2\ldots ,n)$$ (2)

$$Stage\;2:{Η}_{i}=\beta_{0}+\beta_{1}\bar{Y_{j}}+\lambda {Χ}_{k}+\varepsilon_{i}\;(k=1,2\ldots ,n)$$ (3)

where, Hi represents the mental health status of older people; Y_j_ is intergenerational support; and X_k_ represents control variables such as demographic variables, income, place of residence, medical insurance, and pension insurance. The coefficient β_0_ is an intercept item, β_1_ is the regression coefficient of the degree to which the core explanatory variable affects the explanatory variable, λ and γ represent the regression coefficient of X_k_, and ε and θ illustrate the random error term.

## Results

### Descriptive statistics

Table 2 shows the descriptive statistical analysis in this study. In total, 6,647 older people were sampled. The proportion of men and women was balanced, and more than 70% of participants lived in a rural area. The average age was 70.01 years, with most participants concentrated in the age range of 60–70 years. Regarding level of education, most completed primary school. The average value of self-rated health was close to 0: the majority (80.2%) of respondents consider their health status “average or not good,” indicating they are concerned about their own health. If a participant’s total CESD score is greater than or equal to 16 points, that individual is considered to have depression [50]. The mean value of the depression variable in the sample was 8.275, less than 16 rules of thumb. This shows that most participants were in good psychological condition. For intergenerational support, the average value of receiving intergenerational economic support was 0.839, meaning that more than 80% of our sample received intergenerational economic support from their children. However, the average values of emotional support and daily care were only 0.5, indicating that children had less active communication with their older parents and devoted less time to daily care. The descriptive statistics of each variable were reasonable, and no multicollinearity problem was evident after testing.

**Table 2:** Descriptive statistics of variables.

Variable	Obs	Mean	Std.Dev.	Min	Max
Economic support	6647	0.839	0.368	0	1
Emotional support	6647	0.695	0.461	0	1
Caring support	6647	0.534	0.499	0	1
Mental health	6647	8.275	6.896	0	30
Age	6647	70.01	7.533	60	118
Gender	6647	0.5	0.5	0	1
Education	6647	1.968	0.77	1	4
Spouse	6647	0.656	0.475	0	1
Place of residence	6647	0.243	0.429	0	1
Drinking	6647	0.308	0.462	0	1
Smoking	6647	0.042	0.2	0	1
Self-rated health	6647	0.198	0.399	0	1
Pension insurance	6647	0.66	0.474	0	1
Health insurance	6647	0.967	0.18	0	1
Income	6647	2.825	1.786	0	7.591

Table 3 shows the results of the regression analysis. We used an OLS model and stepwise regression analysis to analyze the impact of intergenerational support on the mental health of older people. We first included the core explanatory variables that support the related variables between generations, and then added the control variables. As intergenerational support includes economic support, emotional support, and daily care, three sub-models were constructed. The results of Model 1 showed no significant correlation between economic support and older people’s mental health (β = -0.12, t = -0.54, 95%CI: -0.320, 0.560, P>0.1). This indicates that receiving economic support from their children does not significantly affect their mental health, and verifies Hypothesis 1.

**Table 3:** Intergenerational support and mental health.

	(1)	(2)	(3)
Economic support	0.120		
	(0.225)		
Emotional support		-0.500***	
		(0.178)	
Caring support			-0.437***
			(0.164)
Age	-1.272***	-1.205***	-1.236***
	(0.125)	(0.126)	(0.125)
Gender	-1.387***	-1.432***	-1.409***
	(0.192)	(0.192)	(0.191)
Spouse	0.197	0.234	0.189
	(0.190)	(0.190)	(0.190)
Education	-0.515***	-0.505***	-0.528***
	(0.124)	(0.124)	(0.124)
Place of residence	-1.010***	-1.020***	-1.008***
	(0.230)	(0.229)	(0.229)
Smoking	0.482	0.482	0.488
	(0.409)	(0.409)	(0.409)
Drinking	-0.393**	-0.401**	-0.407**
	(0.191)	(0.191)	(0.191)
Self-rated health	-3.281***	-3.279***	-3.282***
	(0.204)	(0.204)	(0.204)
Pension insurance	0.750***	0.745***	0.774***
	(0.198)	(0.198)	(0.198)
Health insurance	-0.672	-0.643	-0.687
	(0.456)	(0.455)	(0.455)
Income	0.040	0.042	0.041
	(0.048)	(0.048)	(0.048)
_cons	12.786***	13.084***	13.108***
	(0.615)	(0.604)	(0.606)
Obs.	6647	6647	6647
R-squared	0.089	0.090	0.090

Note: ***P<0.01,

**P<0.05,

* P<0.1, in parentheses is the standard error, the same below

Model 2 showed a significant correlation between emotional support and older people’s mental health (β = -0.5, t = -2.8, 95%CI: -0.850, -0.150, P<0.01), i.e., for every unit of children’s increased emotional support, older people’s level of depression decreased by 0.5 units. Furthermore, regular mental consolation from children reduced the occurrence of senile depression and improved mental health. Therefore, Hypothesis 2 was confirmed.

Model 3 showed that daily care significantly influenced older people’s mental health (β = -0.437, t = -2.67, 95%CI: -0.758,-0.116, P<0.01). For every additional unit of daily care from children, the level of depression decreased by 0.437 units. This means that the more time older people received of children’s living care, the less depressed they were and the better their mental health was. Hence, Hypothesis 3 was verified.

In addition, although the relevant control factors are not the main focus of this article, they still affected older people’s mental health. The regression results showed that age, gender, education level, place of residence, self-assessment of health, pension insurance, and whether they drink alcohol or not were significantly correlated with elderly health (P<0.05). These factors can reduce depression and associate with mental health. There was no significant differences regarding spouse, smoking status, and income status (P>0.1), and these did not have a significant impact on the mental health of the older people in our study.

Table 4 shows the endogenous test results. To avoid the causal relationship between intergenerational support and mental health, we follow [55] and use as an instrumental variable whether children work. The results indicate that the first-stage F statistic of the 2LS method is 36.65, which is higher than the rule of thumb of 10 and satisfies the criteria for instrumental variables [56]. In addition, the instrumental variables were significantly correlated with the main explanatory variables for the whole sample. The first-stage regression instrumental variables of intergenerational support of explanatory variables are strongly correlated, and the WALD test of the second-stage regression in the table confirms no problem with endogeneity in the presence of intergenerational support. Moreover, no endogeneity was found for all participants for the three aspects of the intergenerational support model.

**Table 4:** Endogenous test.

	(1) (stage 1)	(2) (stage 2)	(3) (stage 1)	(4) (stage 2)	(5) (stage 1)	(6) (stage 2)
Economic support		-0.482 (1.057)				
Emotional support				-0.628 (1.378)		
Caring support						-0.823 (1.808)
Working	0.419*** (0.031)		0.321*** (0.030)		0.245*** (0.031)	
Control variables	controlled	controlled	controlled	controlled	controlled	controlled
_cons	0.268*** (0.042)	13.162*** (0.896)	0.174*** (0.046)	13.142*** (0.864)	0.355*** (0.051)	13.325*** (1.191)
F-value	39.60		34.87		20.05	
WALD		0.561		0.926		0.830

### Robustness test

#### Replacement variable method

Table 5 shows the results of the analysis of the substitution variable method. In this study, the dependent variable was replaced for the robustness test. Loneliness is a major risk factor for depression and has been shown to predict depressive symptoms and the development of depression. Loneliness is also associated with cognitive impairment [57]. Thus, we replaced the dependent depressive condition with “loneliness.” The results showed that economic support had no notable effect on the loneliness of older people. However, emotional support and daily care had a significant negative impact on their loneliness. In general, the results of robustness test are consistent with the coefficient direction and conclusion of the benchmark regression, and they are robust.

**Table 5:** Alternative variable method.

	(1)	(2)	(3)
	dc017	dc017	dc017
Economic support	0.018		
	(0.014)		
Emotional support		-0.022**	
		(0.011)	
Caring support			-0.042***
			(0.010)
Age	-0.034***	-0.030***	-0.030***
	(0.008)	(0.008)	(0.008)
Gender	-0.026**	-0.029**	-0.028**
	(0.012)	(0.012)	(0.012)
Spouse	-0.128***	-0.126***	-0.129***
	(0.012)	(0.012)	(0.012)
Education	-0.015**	-0.015*	-0.017**
	(0.008)	(0.008)	(0.008)
Place of residence	-0.038***	-0.039***	-0.038***
	(0.014)	(0.014)	(0.014)
Smoking	0.009	0.009	0.010
	(0.025)	(0.025)	(0.025)
Drinking	-0.023**	-0.023**	-0.024**
	(0.012)	(0.012)	(0.012)
Self-rated health	-0.089***	-0.088***	-0.089***
	(0.013)	(0.013)	(0.013)
Pension insurance	0.036***	0.036***	0.039***
	(0.012)	(0.012)	(0.012)
Health insurance	-0.014	-0.011	-0.015
	(0.028)	(0.028)	(0.028)
Income	0.004	0.004	0.004
	(0.003)	(0.003)	(0.003)
_cons	0.397***	0.419***	0.432***
	(0.038)	(0.037)	(0.037)
Obs.	6647	6647	6647
R-squared	0.047	0.047	0.049

#### Propensity score matching inspection

Table 6 displays the propensity score matching average processing effect. We used propensity score matching(PSM) to eliminate possible bias and the self-selection between the intergenerational support of older people and their health status as much as possible to render the analysis results more robust. The results of the PSM average processing effect average treatment effect on the treated (ATT) test showed that after the counterfactual estimation of 1:4 neighbor matching, radius matching (0.01) and kernel matching, the average treatment effect was the same as that of the OLS regression model. The estimation effect also passed the balance test. For economic support, the ATT test value for the treatment group was not noteworthy, indicating that when other factors are excluded, there is no significant correlation between economic support and mental health of older people. For emotional support, the t-test values of the three average processing effects (ATT) of the treatment groups were all at a level of significance of 5% and more, indicating that emotional support could affect older people’s mental health. Finally, for daily care, regardless of the matching method used, the effect of daily care on mental health was significant at the 1% significance level, consistent with the direction and conclusion of the benchmark regression result coefficient. In summary, the results of PSM demonstrate the robustness of the baseline regression results when considering sample selectivity bias.

**Table 6:** Propensity score matching average processing effect.

Interpreted variable	Explanatory variable	Matching method	ATT
Mental health	Intergenerational economic support	Neighbor matching (1:4) Radius match (0.01) Kernel matching	-0.64 0.58 0.64
	Emotional support	Neighbor matching (1:4) Radius match (0.01) Kernel matching	-3.05*** -2.48** -2.48**
	Daily care	Neighbor matching (1:4) Radius match (0.01) Kernel matching	-2.91*** -2.69*** -2.79***

Note:* * *, * *, * indicates that the estimated results were significant at the 1%, 5%, and 10% levels.

### Analysis of heterogeneity

Table 7 shows gender heterogeneity. The results indicate heterogeneity for intergenerational support and the mental health of older people of different genders. Specifically, for economic support, the impact of economic support on women was at a significance level of 5% with an obstructive effect (β = -0.814, t = 2.23, 95%CI: 0.099, 1.528, P<0.05). Economic support had no effect on the mental health of older males, which was consistent with the results of the benchmark regression (β = -0.425, t = 0.278, 95%CI: -0.969, 0.119, P>0.1). Further, the effect of emotional support on women was at a significance level of 5% when showing the effect of improving mental health (β = -0.702, t = 0.250, 95%CI: -1.252, -0.153, P<0.05). Emotional support also had no significant correlation with the mental health of older males (β = -0.319, t = -1.42, 95%CI: -0.7560, 0.123, P>0.1). There was a significant correlation between daily care and the mental health of both genders, consistent with the conclusion of the OLS regression. In addition, women (β = - 0.519, t = -2.06, 95%CI: -1.013, -0.025, P<0.05) and men (β = - 0.348, t = -1.65, 95%CI: -0.762, 0.066, P<0.1) had high significance levels. This verifies Hypothesis 2.

**Table 7:** Gender heterogeneity.

	Mental health (1)	Mental health (2)	Mental health (3)	Mental health (4)	Mental health (5)	Mental health (6)
Economic support	0.814**	-0.425				
	(0.364)	(0.278)				
Emotional support			-0.702**	-0.319		
			(0.280)	(0.225)		
Caring support					-0.519**	-0.348*
					(0.252)	(0.211)
Age	-1.677***	-0.835***	-1.547***	-0.834***	-1.616***	-0.837***
	(0.190)	(0.163)	(0.192)	(0.163)	(0.189)	(0.163)
Spouse	0.234	0.089	0.252	0.060	0.182	0.040
	(0.272)	(0.271)	(0.272)	(0.269)	(0.273)	(0.268)
Education	-0.477**	-0.535***	-0.487***	-0.535***	-0.519***	-0.550***
	(0.187)	(0.163)	(0.187)	(0.163)	(0.188)	(0.163)
Place of residence	-1.429***	-0.570*	-1.500***	-0.527*	-1.461***	-0.531*
	(0.352)	(0.296)	(0.351)	(0.295)	(0.351)	(0.295)
Smoking	0.870	0.299	0.916	0.300	0.934	0.305
	(1.032)	(0.416)	(1.032)	(0.416)	(1.033)	(0.416)
Drinking	0.023	-0.546**	0.041	-0.554***	0.014	-0.553***
	(0.368)	(0.212)	(0.368)	(0.213)	(0.368)	(0.213)
Self-rated health	-3.669***	-2.939***	-3.685***	-2.939***	-3.667***	-2.950***
	(0.327)	(0.254)	(0.327)	(0.254)	(0.327)	(0.254)
Pension insurance	0.467	1.007***	0.501	0.979***	0.533*	1.006***
	(0.312)	(0.249)	(0.311)	(0.249)	(0.311)	(0.249)
Health insurance	-1.124*	0.208	-1.086*	0.170	-1.126*	0.118
	(0.613)	(0.695)	(0.613)	(0.694)	(0.613)	(0.694)
Income	-0.005	0.090	-0.004	0.091	-0.003	0.089
	(0.071)	(0.063)	(0.071)	(0.063)	(0.071)	(0.063)
_cons	13.620***	10.045***	14.588***	9.978***	14.579***	10.035***
	(0.901)	(0.892)	(0.870)	(0.888)	(0.875)	(0.890)
Obs.	3323	3324	3323	3324	3323	3324
R-squared	0.082	0.071	0.082	0.071	0.081	0.071

The possible reasons are as follows: The gender division of labor in families and disadvantaged position of women in the labor market have led to an underestimation of the value of female households and social labor. Older women lack more economic resources than older men and rely heavily on the support of their spouses or adult children [38, 58]. Excessive dependence on older people will have a negative impact on mental health. In addition, men have different roles in the family than women. Older women are more involved in family activities such as taking care of grandchildren and thus have more contact with their children [58]. The happiness of older men stems mostly from social support, and they have less contact with their children [59]. Therefore, the impact of emotional support on the mental health of older men is not as significant as it is for older women.

Table 8 shows the results for age heterogeneity: For all age groups with economic support, there was no significant effect on mental health. Emotional support significantly promoted the mental health of those aged 60–70 and 70–80 years. The effect of emotional support on those aged 80+ years was not significant (β = 0.490, t = 0.82, 95%CI: -0.679, 1.658, P>0.1). Daily care significantly affects the mental health of those aged 70–80 years at the 10% significance level (β = - 0.535, t = -1.81, 95%CI: -1.116, 0.045, P<0.1). However, there is no significant relationship between daily care for those aged 60–70 and 80+ (P> 0.1), confirming Hypothesis 4. There is, however, a positive effect. This may be because most older people aged 60–70 years are likely to participate in intergenerational care [55, 60]. They also participate in more social activities, and have less emotional support and daily dependence on their children. Older people aged 80+ emphasize their ability to maintain their autonomy and independence in later life [61]. Therefore, the impact of daily care on the mental health of younger and older people is not significant.

**Table 8:** Age heterogeneity.

**(1)**						
	Mental health (1)	Mental health (2)	Mental health (3)	Mental health (4)	Mental health (5)	Mental health (6)
Economic support	-0.046	0.104	0.368			
	(0.273)	(0.444)	(0.761)			
Emotional support				-0.480**	-1.136***	0.490
				(0.219)	(0.344)	(0.595)
Gender	-1.289***	-1.956***	-0.668	-1.308***	-2.082***	-0.616
	(0.249)	(0.350)	(0.561)	(0.249)	(0.348)	(0.566)
Spouse	-0.048	-0.132	1.650***	-0.019	-0.075	1.644***
	(0.262)	(0.322)	(0.546)	(0.261)	(0.321)	(0.545)
Education	-0.818***	-0.375*	0.031	-0.816***	-0.341	0.001
	(0.161)	(0.227)	(0.361)	(0.161)	(0.226)	(0.362)
Place of residence	-1.160***	-0.979**	0.005	-1.157***	-0.967**	0.013
	(0.301)	(0.412)	(0.653)	(0.300)	(0.409)	(0.653)
Smoking	0.471	0.268	0.871	0.462	0.280	0.837
	(0.552)	(0.743)	(1.023)	(0.552)	(0.741)	(1.024)
Drinking	-0.439*	-0.719**	1.142*	-0.449*	-0.743**	1.154**
	(0.249)	(0.343)	(0.584)	(0.249)	(0.342)	(0.584)
Self-rated health	-3.658***	-3.377***	-0.359	-3.657***	-3.372***	-0.371
	(0.258)	(0.382)	(0.635)	(0.258)	(0.381)	(0.634)
Pension insurance	1.138***	0.816**	-0.679	1.125***	0.793**	-0.646
	(0.268)	(0.347)	(0.536)	(0.267)	(0.345)	(0.537)
Health insurance	-0.510	-0.697	-0.235	-0.508	-0.613	-0.282
	(0.720)	(0.735)	(0.983)	(0.718)	(0.732)	(0.982)
Income	0.044	0.003	0.159	0.046	-0.002	0.156
	(0.062)	(0.087)	(0.135)	(0.062)	(0.087)	(0.135)
_cons	11.931***	11.312***	5.362***	12.183***	12.185***	5.350***
	(0.825)	(0.950)	(1.372)	(0.817)	(0.910)	(1.250)
Obs.	3706	2085	856	3706	2085	856
R-squared	0.114	0.091	0.023	0.115	0.095	0.024
**(2)**						
	Mental health (1)		Mental health (2)		Mental health (3)	
Caring support	-0.295		-0.535*		-0.720	
	(0.212)		(0.296)		(0.482)	
Gender	-1.302***		-1.975***		-0.666	
	(0.249)		(0.347)		(0.558)	
Spouse	-0.054		-0.158		1.603***	
	(0.261)		(0.322)		(0.545)	
Education	-0.824***		-0.395*		-0.002	
	(0.161)		(0.227)		(0.361)	
Place of residence	-1.139***		-0.974**		-0.051	
	(0.300)		(0.410)		(0.654)	
Smoking	0.462		0.282		0.926	
	(0.552)		(0.743)		(1.023)	
Drinking	-0.445*		-0.743**		1.102*	
	(0.249)		(0.343)		(0.584)	
Self rated health	-3.657***		-3.399***		-0.355	
	(0.258)		(0.382)		(0.634)	
Pension insurance	1.152***		0.825**		-0.654	
	(0.267)		(0.346)		(0.535)	
Health insurance	-0.533		-0.736		-0.227	
	(0.719)		(0.733)		(0.981)	
Income	0.043		0.006		0.161	
	(0.062)		(0.087)		(0.135)	
_cons	12.076***		11.805***		6.211***	
	(0.817)		(0.908)		(1.220)	
Obs.	3706		2085		856	
R-squared	0.115		0.092		0.026	

Table 9 shows the results for urban and rural heterogeneity. No significant correlation was found between economic support and mental health of older people, consistent with the benchmark regression results. Compared to urban areas, emotional support (β = - 0.570, t = -2.73, 95% CI: -0.979, -0.161, P<0.01) and daily care (β = - 0.408, t = -2.21, 95%CI: -0.786, -0.030, P<0.05) more significantly affected the mental health of older people in rural areas, validating Hypothesis 4.

**Table 9:** Urban-rural heterogeneity.

	Mental health (1)	Mental health (2)	Mental health (3)	Mental health (4)	Mental health (5)	Mental health (6)
Economic support	0.066	0.050				
	(0.282)	(0.359)				
Emotional support			-0.570***	-0.322		
			(0.209)	(0.340)		
Caring support					-0.408**	-0.410
					(0.193)	(0.307)
Age	-1.554***	-0.406*	-1.486***	-0.359	-1.518***	-0.393*
	(0.147)	(0.233)	(0.148)	(0.235)	(0.147)	(0.231)
Gender	-1.595***	-0.873**	-1.654***	-0.883**	-1.611***	-0.896**
	(0.227)	(0.360)	(0.227)	(0.360)	(0.226)	(0.360)
Spouse	0.148	0.285	0.189	0.298	0.141	0.263
	(0.223)	(0.360)	(0.222)	(0.360)	(0.222)	(0.360)
Education	-0.512***	-0.400*	-0.490***	-0.405*	-0.520***	-0.417*
	(0.152)	(0.213)	(0.152)	(0.213)	(0.152)	(0.213)
Smoking	-0.045	1.348**	-0.026	1.323**	-0.031	1.338**
	(0.513)	(0.657)	(0.513)	(0.657)	(0.513)	(0.656)
Drinking	-0.260	-0.810**	-0.276	-0.806**	-0.276	-0.816**
	(0.225)	(0.359)	(0.225)	(0.359)	(0.225)	(0.359)
Self-rated health	-3.122***	-3.636***	-3.122***	-3.632***	-3.127***	-3.629***
	(0.246)	(0.357)	(0.246)	(0.357)	(0.246)	(0.356)
Pension insurance	0.593**	1.144***	0.575**	1.148***	0.605**	1.182***
	(0.236)	(0.365)	(0.236)	(0.364)	(0.236)	(0.365)
Health insurance	-0.531	-1.624	-0.501	-1.611	-0.538	-1.707
	(0.497)	(1.224)	(0.496)	(1.224)	(0.496)	(1.225)
Income	0.063	0.013	0.063	0.015	0.062	0.016
	(0.061)	(0.074)	(0.061)	(0.074)	(0.061)	(0.074)
_cons	13.273***	10.936***	13.570***	11.118***	13.530***	11.301***
	(0.697)	(1.445)	(0.681)	(1.438)	(0.682)	(1.450)
Obs.	5034	1613	5034	1613	5034	1613
R-squared	0.075	0.097	0.077	0.097	0.076	0.098

This may be because the norm of “raising children to prevent aging” is more concrete in rural than in urban areas. Older people in rural areas have fewer social activities and depend heavily on their children [62]. They are also more inclined to confide in their children. Due to the rise of modern civilized lifestyle in cities and changes in the concept of intergenerational families, older people in cities are more independent than those in rural areas, with low dependence on the emotional support of family members [13]. In addition, inter-family relations in rural areas are reflected in the feedback model in which children support their parents and are more involved in intergenerational care. Therefore, the impact on emotional support and daily care is more significant in rural than in urban areas.

## Discussion

This study explored the impact of intergenerational support on older people’s mental health. Based on an OLS regression analysis, we also performed an endogenous test, variable substitution, and PSM to analyze the robustness of the results of the CHARLS data. Considering the differences in the effects of intergenerational support on the mental health status of older people of different ages, genders, and places of residence, this study also conducted a heterogeneity regression. In the context of aging, it is hoped that our results will draw family members’ attention to improving intergenerational support, enhance older people’s mental health, and provide evidence for policy makers.

First, the regression results indicate that intergenerational economic support has no significant impact on older people’s mental health. Aligned with Maslow’s hierarchy of needs theory, many scholars support this viewpoint [37, 38]. Relative to China’s developing economy, the living standards of older people satisfy their physiological needs, and economic support may no longer be an important factor affecting their mental health. In addition, economic support is reflected more in the help of physical health and cannot replace the feeling of loneliness prevalent among older people in their later years. Thus, mutual communication and emotional connection are more important in addressing depression among older people [63].

Second, our study shows that emotional support and daily care can help ease depression among older people, consequently promoting mental health. These conclusions echo those of other studies [23–25]. This confirms the positive effect of social exchange theory and is consistent with the hypothesis of the feedback theory. The parenting behavior of parents receives feedback later. Emotional support and daily care are manifestations of “support” behavior, which is consistent with traditional Chinese filial piety culture and expectations [64]. In Western culture, the differences between social exchange patterns and family relationships are not more dependent on children; thus, intergenerational support does not significantly influence older people’s mental health. For example, a national health and ageing survey in Mexico showed that people aged over 60 years often live alone and rely on their own pensions and other informal support to satisfy their physical and mental needs [60] A US study of 403 parents aged 60+ years in Washington State also suggested that interactions with relatives had no major effect on the emotional well-being of older adults [65]. Older people living with their children have stronger intergenerational relationships and are relatively less likely to suffer from depression, which benefits family intergenerational unity [66, 67]. Emotional support and daily care reflect a sense of belonging, concern, and support for older people, which satisfies their psychological health needs [68].

Finally, our study included key baseline demographic factors of intergenerational support (age, gender, place of residence) as variables for the heterogeneity analysis. The results confirmed the significant effect of economic support on the mental health of older women, but this was an obstacle. For each additional unit of economic support, the depressive condition increased by 0.814 units. Thus, intergenerational economic support is not conducive to the mental health of older women, and has no impact on that of older men. This is consistent with previous research findings. A possible reason is that in Chinese family relationships, the division of roles, namely that “men lead the outside and women the inside,” means that older men receive a higher pension upon retirement [68]. In contrast, older women have limited resources and rely more on their children’s economic assistance [69]. However, excessive benefits bring guilt and psychological pressure, leading to depression. Another interesting finding is that emotional support gradually weakens its impact on the mental health of older people as they age. From a demand perspective, as age increases, older people and their state of health decline, while symptoms of depression increase. Emotional support cannot significantly affect the depressive state on its own [70]. On the other hand, older people may emphasize different aspects of life [61]. They may set out to realize their self-worth, such as providing intergenerational support to their children [31], rather than excessively benefiting from others. They may emphasize independence and autonomy in their later years of life. Here, the impact of emotional support and daily care on the mental health of older people in rural areas is stronger than on those in urban regions. Although discussed earlier, we highlight imbalanced resource conditions and the phenomenon of older people living alone in rural areas renders them more prone to depression [43]. A formal or informal social support system is needed to help meet the daily care and medical services of older people.

Through these results, we provide empirical support for family community relations and the introduction of government policies from a health perspective. At the family level, in the context of aging, family factors occupy an important position in China’s current pension system. Providing more emotional support and daily care to all older groups, rather than just economic transfer, means that children and the government should pay more attention to the higher-level needs of older people [71]. We should further carry forward the traditional filial piety culture, give play to the traditional ethical role of intergenerational responsibility in providing for older people, and reshape the intergenerational responsibility ethics of pension services [72]. Furthermore, we must improve children’s awareness of supporting parents, focus on parents’ mental health, and improve their living standards. Other actions are to implement activities promoting respect and love of older people, encourage children to be filial to them, establish good intergenerational relations with them, and encourage children’s’ support for their parents. Finally, we must remind children to provide economic support and help as well as emotional support and daily care for older people according to their age, gender, residence, and other mental health needs, which will contribute to the balanced development of their mental health.

Intergenerational support is more inclined toward the elderly, female groups, and rural areas to minimize the differences in health effects among vulnerable older people. At the community level, it can guide the practice of community work. Community work can help establish a good social environment and encourage young people to respect and be filial to older adults. Therefore, community work interventions should be implemented to improve family relationships. Furthermore, social participation can improve older people’s mental health [73], as community activities can compensate for the loneliness caused by the lack of intergenerational relations.

Of course, home care for older people can be supplemented with community care or the community could provide door-to-door services for older people in rural areas or those with disabilities. Nationally, when formulating policies, the government should incorporate intergenerational support into the healthy aging policy and give full play to the role of the family pension function. To this end, it should ensure the provision of social support, emotional support, and adequate daily care conditions, and constantly improve the endowment insurance system. Furthermore, the responsibility ethics of children to support their parents should be cultivated [72]. If the working hours of children are adjusted, they would be able to visit their relatives. Adult children are encouraged to give older people more intergenerational support. We should also continue to improve the multi-level endowment insurance system and medical insurance system, build a diversified endowment service supply system based on home care and supported by communities and supplemented by institutions, and realize active aging.

## Conclusions

Our study showed that economic support in intergenerational support has no significant impact on older people’s mental health, and that emotional support and daily care can reduce their depression and associate with mental health. Various tests confirmed the reliability of this conclusion. However, economic support from children is not everything. Parents should also be given more emotional comfort. Finally, the government should improve the medical security system and elderly care service system, and provide policy support to enhance older people’s mental health.

### Limitation

There are several limitations in this study. First, the cross-sectional study only analyzed the relationship between intergenerational support and mental health of older people, and cannot clarify the causal relationship between the two. Therefore, an analysis of longitudinal data is needed. Second, although the 2SLS and PSM addressed the endogeneity issue to some extent, it was not fully resolved. Further research should consider potential reverse causal relationships. Finally, as CHARLS is second-hand data, we could not use more objective indicators to represent the relevant variables. In the next step, longitudinal survey data should be used to clarify the causal relationship between the data, and a more comprehensive analysis is needed of the relationship between intergenerational support and older people’s mental health. Furthermore, more objective conceptual measures such as physiological indicators or measurements that combine multiple data sources like economic income, number of meetings with children, and so on should be combined to improve the reliability of the results.

## Supporting information

S1 Dataset (RAR)

S2 Dataset (ZIP)

S3 Dataset (RAR)

S1 File (PDF)

S2 File (DOCX)
