# Supplementary material for: Influence of intergenerational support on the mental health of older people in China
Source: PLoS One. 2024 Apr 18;19(4):e0299986. doi: 10.1371/journal.pone.0299986 (PMC11025908; doi:10.1371/journal.pone.0299986)
Supplement: S2 File — (DOCX) [file pone.0299986.s005.docx]

**Here are the specifics of regression analysis:**

**Table 1. Regression analysis of economic support and mental health**

| Mentalhealth | Coef. | | St.Err | | t-value | p-value | Sig. | [95%conf.interval] |
| --- | --- | --- | --- | --- | --- | --- | --- | --- |
| Economic support | 0.120 | | 0.225 | | 0.54 | 0.593 |  | -0.3200 0.5602 |
| Age | -1.272 | | 0.125 | | -10.16 | 0.000 | *** | -1.5175 -1.0264 |
| Gender | -1.387 | | 0.192 | | -7.23 | 0.000 | *** | -1.7633 -1.0114 |
| Spouse | 0.197 | | 0.190 | | 1.04 | 0.300 |  | -0.1757 0 .5704 |
| Education | -0.515 | | 0.124 | | -4.15 | 0.000 | *** | -0.7585 0.2715 |
| Place of residence | -1.010 | | 0.230 | | -4.40 | 0.000 | *** | -1.4598 -0.5595 |
| Smoking | 0.482 | | 0.409 | | 1.18 | 0.239 |  | -0.3200 1.2843 |
| Drinking | -0.393 | | 0.191 | | -2.05 | 0.040 | ** | 0.7678 -0.0175 |
| SelfratedHealth | -3.281 | | 0.204 | | -16.05 | 0.000 | *** | -3.6819 -2.8802 |
| PensionInsurance | 0.750 | | 0.198 | | 3.78 | 0.000 | *** | 0.3612 1.1391 |
| HealthInsurance | -0.672 | | 0.456 | | -1.48 | 0.140 |  | -1.5650 0.2212 |
| Income | 0.040 | | 0.048 | | 0.85 | 0.397 |  | -0.0529 0.1336 |
| _cons | 12.786 | | 0.615 | | 20.78 | 0.000 | *** | 11.5803 13.9924 |
|  | | | | | | | |  |
| Mean dependent var | | 8.275 | | SD dependent var | | | 6.896 |  |
| R-squared | | 0.089 | | Number of obs | | | 6647.000 |  |
| F-test | | 54.090 | | Prob > F | | | 0.000 |  |
| Akaike crit. (AIC) | | 43937.423 | | Bayesian crit. (BIC) | | | 44025.848 |  |
|  | | | | | | | |  |
| *** p<0.01, ** p<0.05, * p<0.1 | | | | | | | |  |

**Table 2. Regression results of emotional support and mental health**

| Mentalhealth | Coef. | | St.Err | t-value | p-value | Sig. | [95%conf.interval] |
| --- | --- | --- | --- | --- | --- | --- | --- |
| Emotional Support | -0.500 | | 0.178 | -2.80 | 0.005 | *** | -0.8500 -0.1504 |
| Age | -1.205 | | 0.126 | -9.57 | 0.000 | *** | -1.4520 -0.9584 |
| Gender | -1.432 | | 0.192 | -7.48 | 0.000 | *** | -1.8081 -1.0569 |
| Spouse | 0.234 | | 0.190 | 1.23 | 0.218 |  | -0.1381 0.6064 |
| Education | -0.505 | | 0.124 | -4.07 | 0.000 | *** | -0.7485 -0.2616 |
| Place of residence | -1.020 | | 0.229 | -4.46 | 0.000 | *** | -1.4684 -0.5717 |
| Smoking | 0.482 | | 0.409 | 1.18 | 0.238 |  | -0.3194 1.2840 |
| Drinking | -0.401 | | 0.191 | -2.10 | 0.036 | ** | -0.7761 -0.0261 |
| SelfratedHealth | -3.279 | | 0.204 | -16.04 | 0.000 | *** | -3.6795 -2.8782 |
| PensionInsurance | 0.745 | | 0.198 | 3.77 | 0.000 | *** | 0.3573 1.1333 |
| HealthInsurance | -0.643 | | 0.455 | -1.41 | 0.158 |  | -1.5351 0.2492 |
| Income | 0.042 | | 0.048 | 0.88 | 0.380 |  | -0.0515 0.1349 |
| _cons | 13.084 | | 0.604 | 21.66 | 0.000 | *** | 11.9003 14.2686 |
|  | | | | | | |  |
| Mean dependent var | | 8.275 | | SD dependent var | | 6.896 |  |
| R-squared | | 0.090 | | Number of obs | | 6647.000 |  |
| F-test | | 54.783 | | Prob > F | | 0.000 |  |
| Akaike crit. (AIC) | | 43929.841 | | Bayesian crit. (BIC) | | 44018.266 |  |
|  | | | | | | |  |
| *** p<0.01, ** p<0.05, * p<0.1 | | | | | | |  |

**Table 3. Regression analysis of caring suppor and mental health**

| Mentalhealth | Coef. | | St.Err | | t-value | p-value | Sig. | | [95%conf.interval] |
| --- | --- | --- | --- | --- | --- | --- | --- | --- | --- |
| CaringSuppor | -0.437 | | 0.164 | | -2.67 | 0.008 | *** | | -0.7583 -0.1159 |
| Age | -1.236 | | 0.125 | | -9.92 | 0.000 | *** | | -1.4806 -0.9922 |
| Gender | -1.409 | | 0.191 | | -7.37 | 0.000 | *** | | -1.7837 -1.0340 |
| Spouse | 0.189 | | 0.190 | | 1.00 | 0.318 |  | | -.18245 0.5613 |
| Education | -0.528 | | 0.124 | | -4.25 | 0.000 | *** | | -0.7713 -0.2842 |
| Place of residence | -1.008 | | 0.229 | | -4.41 | 0.000 | *** | | -1.4562 -0.5592 |
| Smoking | 0.488 | | 0.409 | | 1.19 | 0.233 |  | | -0.3139 1.2896 |
| Drinking | -0.407 | | 0.191 | | -2.13 | 0.033 | ** | | -0.7825 -0.0323 |
| SelfratedHealth | -3.282 | | 0.204 | | -16.06 | 0.000 | *** | | -3.6831 -2.8818 |
| PensionInsurance | 0.774 | | 0.198 | | 3.91 | 0.000 | *** | | 0.3861 1.1624 |
| HealthInsurance | -0.687 | | 0.455 | | -1.51 | 0.131 |  | | -1.5792 0.2053 |
| Income | 0.041 | | 0.048 | | 0.85 | 0.392 |  | | -0.0525 0.1339 |
| _cons | 13.108 | | 0.606 | | 21.63 | 0.000 | *** | | 11.9198 14.2953 |
|  | | | | | | | | |  |
| Mean dependent var | | 8.275 | | SD dependent var | | | | 6.896 |  |
| R-squared | | 0.090 | | Number of obs | | | | 6647.000 |  |
| F-test | | 54.715 | | Prob > F | | | | 0.000 |  |
| Akaike crit. (AIC) | | 43930.582 | | Bayesian crit. (BIC) | | | | 44019.007 |  |
|  | | | | | | | | |  |
| *** p<0.01, ** p<0.05, * p<0.1 | | | | | | | | |  |

**Table 4. Regression analysis of female economic support and mental health**

| Mentalhealth | Coef. | St.Err | | t-value | p-value | Sig. | [95%conf.interval] |
| --- | --- | --- | --- | --- | --- | --- | --- |
| Economic support | 0.814 | 0.364 | | 2.23 | 0.026 | ** | 0.0995 1.5278 |
| Age | -1.677 | 0.190 | | -8.81 | 0.000 | *** | -2.0500 -1.3035 |
| Spouse | 0.234 | 0.272 | | 0.86 | 0.390 |  | -0.2990 0.7661 |
| Education | -0.477 | 0.187 | | -2.54 | 0.011 | ** | -0.8442 -0.1095 |
| Place of residence | -1.429 | 0.352 | | -4.06 | 0.000 | *** | -2.1195 -0.7387 |
| Smoking | 0.870 | 1.032 | | 0.84 | 0.399 |  | -1.1540 2.8940 |
| Drinking | 0.023 | 0.368 | | 0.06 | 0.950 |  | -0.6983 0.7449 |
| SelfratedHealth | -3.669 | 0.327 | | -11.23 | 0.000 | *** | -4.3096 -3.0289 |
| PensionInsurance | 0.467 | 0.312 | | 1.50 | 0.135 |  | -0.1450 1.0785 |
| HealthInsurance | -1.124 | 0.613 | | -1.83 | 0.067 | * | -2.3261 0.0790 |
| Income | -0.005 | 0.071 | | -0.07 | 0.943 |  | -0.1444 0.1342 |
| _cons | 13.620 | 0.901 | | 15.11 | 0.000 | *** | 11.8527 15.38780 |
|  | | | | | | |  |
| Mean dependent var | 9.190 | | SD dependent var | | | 7.379 |  |
| R-squared | 0.082 | | Number of obs | | | 3323.000 |  |
| F-test | 26.724 | | Prob > F | | | 0.000 |  |
| Akaike crit. (AIC) | 22453.313 | | Bayesian crit. (BIC) | | | 22526.617 |  |
|  | | | | | | |  |
| *** p<0.01, ** p<0.05, * p<0.1 | | | | | | |  |

**Table 5. Regression analysis of male economic support and mental health**

| Mentalhealth | Coef. | | St.Err | | t-value | p-value | Sig. | [95%conf.interval] |
| --- | --- | --- | --- | --- | --- | --- | --- | --- |
| Economic support | -0.425 | | 0.278 | | -1.53 | 0.126 |  | -0.9690 0.1193 |
| Age | -0.835 | | 0.163 | | -5.12 | 0.000 | *** | -1.1548 -0.5154 |
| Spouse | 0.089 | | 0.271 | | 0.33 | 0.742 |  | -0.4424 0.6210 |
| Education | -0.535 | | 0.163 | | -3.28 | 0.001 | *** | -0.8551 -0.2151 |
| Place of residence | -0.570 | | 0.296 | | -1.93 | 0.054 | * | -1.15055 0.0099 |
| Smoking | 0.299 | | 0.416 | | 0.72 | 0.473 |  | -0.5168 1.1139 |
| Drinking | -0.546 | | 0.212 | | -2.57 | 0.010 | ** | -0.9625 -0.1294 |
| SelfratedHealth | -2.939 | | 0.254 | | -11.59 | 0.000 | *** | -3.4363 -2.4415 |
| PensionInsurance | 1.007 | | 0.249 | | 4.04 | 0.000 | *** | 0.5180 1.4959 |
| HealthInsurance | 0.208 | | 0.695 | | 0.30 | 0.765 |  | -1.1550 1.5716 |
| Income | 0.090 | | 0.063 | | 1.44 | 0.150 |  | -0.0327 0.2134 |
| _cons | 10.045 | | 0.892 | | 11.26 | 0.000 | *** | 8.2962 11.7931 |
|  | | | | | | | |  |
| Mean dependent var | | 7.360 | | SD dependent var | | | 6.245 |  |
| R-squared | | 0.071 | | Number of obs | | | 3324.000 |  |
| F-test | | 23.009 | | Prob > F | | | 0.000 |  |
| Akaike crit. (AIC) | | 21389.024 | | Bayesian crit. (BIC) | | | 21462.331 |  |
|  | | | | | | | |  |
| *** p<0.01, ** p<0.05, * p<0.1 | | | | | | | |  |

| Mentalhealth | Coef. | | St.Err | | t-value | p-value | | Sig. | [95%conf.interval] |
| --- | --- | --- | --- | --- | --- | --- | --- | --- | --- |
| EmotionalSupport | -0.702 | | 0.280 | | -2.50 | 0.012 | | ** | -1.2518 -0.1526 |
| Age | -1.547 | | 0.192 | | -8.05 | 0.000 | | *** | -1.9244 -1.1705 |
| Spouse | 0.252 | | 0.272 | | 0.93 | 0.354 | |  | -0.2810 0.7843 |
| Education | -0.487 | | 0.187 | | -2.60 | 0.009 | | *** | -0.8544 -0.1205 |
| Place of residence | -1.500 | | 0.351 | | -4.27 | 0.000 | | *** | -2.1882 -0.8116 |
| Smoking | 0.916 | | 1.032 | | 0.89 | 0.375 | |  | -1.1080 2.9391 |
| Drinking | 0.041 | | 0.368 | | 0.11 | 0.912 | |  | -0.6804 0.7619 |
| SelfratedHealth | -3.685 | | 0.327 | | -11.28 | 0.000 | | *** | -4.3259 -3.0451 |
| PensionInsurance | 0.501 | | 0.311 | | 1.61 | 0.108 | | * | -0.1091 1.1108 |
| HealthInsurance | -1.086 | | 0.613 | | -1.77 | 0.077 | | * | -2.2880 0.1166 |
| Income | -0.004 | | 0.071 | | -0.06 | 0.952 | |  | -0.1436 0.1350 |
| _cons | 14.588 | | 0.870 | | 16.77 | 0.000 | | *** | 12.8827 16.2934 |
|  | | | | | | | | |  |
| Mean dependent var | | 9.190 | | SD dependent var | | | 7.379 | |  |
| R-squared | | 0.082 | | Number of obs | | | 3323.000 | |  |
| F-test | | 26.851 | | Prob > F | | | 0.000 | |  |
| Akaike crit. (AIC) | | 22452.027 | | Bayesian crit. (BIC) | | | 22525.331 | |  |
|  | | | | | | | | |  |
| *** p<0.01, ** p<0.05, * p<0.1 | | | | | | | | |  |

**Table 6. Regression results of female emotional support and mental health**

**Table 7. Regression results of male emotional support and mental health**

| Mentalhealth | Coef. | | St.Err | | t-value | p-value | | Sig. | [95%conf.interval] |
| --- | --- | --- | --- | --- | --- | --- | --- | --- | --- |
| EmotionalSupport | -0.319 | | 0.225 | | -1.42 | 0.157 | |  | -0.7560 0.1225 |
| Age | -0.834 | | 0.163 | | -5.10 | 0.000 | | *** | -1.1542 -0.5133 |
| Spouse | 0.060 | | 0.269 | | 0.22 | 0.825 | |  | -0.4679 0.5870 |
| Education | -0.535 | | 0.163 | | -3.28 | 0.001 | | *** | -0.8552 -0.2151 |
| Place of residence | -0.527 | | 0.295 | | -1.79 | 0.074 | | * | -1.1055 0.0511 |
| Smoking | 0.300 | | 0.416 | | 0.72 | 0.470 | |  | -0.5149 1.1158 |
| Drinking | -0.554 | | 0.213 | | -2.61 | 0.009 | | *** | -0.9708 -0.1373 |
| SelfratedHealth | -2.939 | | 0.254 | | -11.58 | 0.000 | | *** | -3.4364 -2.4415 |
| PensionInsurance | 0.979 | | 0.249 | | 3.93 | 0.000 | | *** | 0.4911 1.4678 |
| HealthInsurance | 0.170 | | 0.694 | | 0.24 | 0.807 | |  | -1.1919 1.5312 |
| Income | 0.091 | | 0.063 | | 1.45 | 0.147 | |  | -0.0321 0.2142 |
| _cons | 9.978 | | 0.888 | | 11.23 | 0.000 | | *** | 8.2365 11.7190 |
|  | | | | | | | | |  |
| Mean dependent var | | 7.360 | | SD dependent var | | | 6.245 | |  |
| R-squared | | 0.071 | | Number of obs | | | 3324.000 | |  |
| F-test | | 22.976 | | Prob > F | | | 0.000 | |  |
| Akaike crit. (AIC) | | 21389.363 | | Bayesian crit. (BIC) | | | 21462.670 | |  |
|  | | | | | | | | |  |
| *** p<0.01, ** p<0.05, * p<0.1 | | | | | | | | |  |

**Table 8. Regression results of female caring support and mental health**

| Mentalhealth | Coef. | | St.Err | | t-value | p-value | | Sig. | [95%conf.interval] |
| --- | --- | --- | --- | --- | --- | --- | --- | --- | --- |
| CaringSuppor | -0.519 | | 0.252 | | -2.06 | 0.040 | | ** | -1.0128 -0.0245 |
| Age | -1.616 | | 0.189 | | -8.53 | 0.000 | | *** | -1.9879 -1.2449 |
| Spouse | 0.182 | | 0.273 | | 0.67 | 0.504 | |  | -0.3528 0.7173 |
| Education | -0.519 | | 0.188 | | -2.77 | 0.006 | | *** | -0.8864 -0.1508 |
| Place of residence | -1.461 | | 0.351 | | -4.16 | 0.000 | | *** | -2.1505 -0.7725 |
| Smoking | 0.934 | | 1.033 | | 0.90 | 0.366 | |  | -1.0907 2.9582 |
| Drinking | 0.014 | | 0.368 | | 0.04 | 0.971 | |  | -0.7085 0.7357 |
| SelfratedHealth | -3.667 | | 0.327 | | -11.23 | 0.000 | | *** | -4.3074 -3.0265 |
| PensionInsurance | 0.533 | | 0.311 | | 1.71 | 0.087 | | * | -0.0775 1.1427 |
| HealthInsurance | -1.126 | | 0.613 | | -1.83 | 0.067 | | * | -2.3285 0.0771 |
| Income | -0.003 | | 0.071 | | -0.04 | 0.964 | |  | -0.1425 0.1362 |
| _cons | 14.579 | | 0.875 | | 16.67 | 0.000 | | *** | 12.8644 16.2944 |
|  | | | | | | | | |  |
| Mean dependent var | | 9.190 | | SD dependent var | | | 7.379 | |  |
| R-squared | | 0.081 | | Number of obs | | | 3323.000 | |  |
| F-test | | 26.650 | | Prob > F | | | 0.000 | |  |
| Akaike crit. (AIC) | | 22454.068 | | Bayesian crit. (BIC) | | | 22527.371 | |  |
|  | | | | | | | | |  |
| *** p<0.01, ** p<0.05, * p<0.1 | | | | | | | | |  |

**Table 9. Regression results of male caring support and mental health**

| Mentalhealth | Coef. | | St.Err | | t-value | p-value | | Sig. | [95%conf.interval] |
| --- | --- | --- | --- | --- | --- | --- | --- | --- | --- |
| CaringSuppor | -0.348 | | 0.211 | | -1.65 | 0.099 | | * | -0.7621 0.0660 |
| Age | -0.837 | | 0.163 | | -5.14 | 0.000 | | *** | -1.1561 -0.5181 |
| Spouse | 0.040 | | 0.268 | | 0.15 | 0.880 | |  | -0.4850 0.5660 |
| Education | -0.550 | | 0.163 | | -3.37 | 0.001 | | *** | -0.8699 -0.2303 |
| Place of residence | -0.531 | | 0.295 | | -1.80 | 0.072 | | * | -1.1096 0.0467 |
| Smoking | 0.305 | | 0.416 | | 0.73 | 0.464 | |  | -0.5106 1.1199 |
| Drinking | -0.553 | | 0.213 | | -2.60 | 0.009 | | *** | -0.9697 -0.1364 |
| SelfratedHealth | -2.950 | | 0.254 | | -11.63 | 0.000 | | *** | -3.4472 -2.4526 |
| PensionInsurance | 1.006 | | 0.249 | | 4.03 | 0.000 | | *** | 0.5169 1.4944 |
| HealthInsurance | 0.118 | | 0.694 | | 0.17 | 0.865 | |  | -1.2440 1.4793 |
| Income | 0.089 | | 0.063 | | 1.42 | 0.157 | |  | -0.0341 0.2120 |
| _cons | 10.035 | | 0.890 | | 11.27 | 0.000 | | *** | 8.2893 11.7802 |
|  | | | | | | | | |  |
| Mean dependent var | | 7.360 | | SD dependent var | | | 6.245 | |  |
| R-squared | | 0.071 | | Number of obs | | | 3324.000 | |  |
| F-test | | 23.045 | | Prob > F | | | 0.000 | |  |
| Akaike crit. (AIC) | | 21388.650 | | Bayesian crit. (BIC) | | | 21461.957 | |  |
|  | | | | | | | | |  |
| *** p<0.01, ** p<0.05, * p<0.1 | | | | | | | | |  |

**Table 10. Regression analysis of economic support and 60-70 geriatric mental health**

| Mentalhealth | Coef. | | St.Err | | t-value | p-value | Sig. | [95%conf.interval] |
| --- | --- | --- | --- | --- | --- | --- | --- | --- |
| Economic support | -0.046 | | 0.273 | | -0.17 | 0.867 |  | -0.5810 0.4892 |
| Gender | -1.289 | | 0.249 | | -5.17 | 0.000 | *** | -1.7784 -0.8002 |
| Spouse | -0.048 | | 0.262 | | -0.18 | 0.854 |  | -0.5611 0.4650 |
| Education | -0.818 | | 0.161 | | -5.08 | 0.000 | *** | -1.1329 -0.5022 |
| Place of residence | -1.160 | | 0.301 | | -3.85 | 0.000 | *** | -1.7508 -0.5696 |
| Smoking | 0.471 | | 0.552 | | 0.85 | 0.394 |  | -0.6117 1.5534 |
| Drinking | -0.439 | | 0.249 | | -1.76 | 0.078 | * | -0.9275 0.0488 |
| SelfratedHealth | -3.658 | | 0.258 | | -14.18 | 0.000 | *** | -4.1639 -3.1522 |
| PensionInsurance | 1.138 | | 0.268 | | 4.25 | 0.000 | *** | 0.6129 1.6623 |
| HealthInsurance | -0.510 | | 0.720 | | -0.71 | 0.479 |  | -1.9209 0.9008 |
| Income | 0.044 | | 0.062 | | 0.71 | 0.476 |  | -0.0770 0.1650 |
| _cons | 11.931 | | 0.825 | | 14.47 | 0.000 | *** | 10.3140 13.5471 |
|  | | | | | | | |  |
| Mean dependent var | | 8.710 | | SD dependent var | | | 6.822 |  |
| R-squared | | 0.114 | | Number of obs | | | 3706.000 |  |
| F-test | | 43.288 | | Prob > F | | | 0.000 |  |
| Akaike crit. (AIC) | | 24323.294 | | Bayesian crit. (BIC) | | | 24397.906 |  |
|  | | | | | | | |  |
| *** p<0.01, ** p<0.05, * p<0.1 | | | | | | | |  |

**Table 11. Regression analysis of economic support and 70-80 geriatric mental health**

| Mentalhealth | Coef. | | St.Err | | t-value | p-value | | Sig. | [95%conf.interval] |
| --- | --- | --- | --- | --- | --- | --- | --- | --- | --- |
| Economic support | 0.104 | | 0.444 | | 0.23 | 0.815 | |  | -0.7662 0.9738 |
| Gender | -1.956 | | 0.350 | | -5.59 | 0.000 | | *** | -2.6422 -1.2693 |
| Spouse | -0.132 | | 0.322 | | -0.41 | 0.682 | |  | -0.7642 0.5001 |
| Education | -0.375 | | 0.227 | | -1.65 | 0.099 | | * | -0.8195 0.0670 |
| Place of residence | -0.979 | | 0.412 | | -2.38 | 0.018 | | ** | -1.7867 -0.1707 |
| Smoking | 0.268 | | 0.743 | | 0.36 | 0.718 | |  | -1.1899 1.7260 |
| Drinking | -0.719 | | 0.343 | | -2.10 | 0.036 | | ** | -1.3916 -0.0462 |
| SelfratedHealth | -3.377 | | 0.382 | | -8.85 | 0.000 | | *** | -4.1251 -2.6280 |
| PensionInsurance | 0.816 | | 0.347 | | 2.35 | 0.019 | | ** | 0.13641 1.4957 |
| HealthInsurance | -0.697 | | 0.735 | | -0.95 | 0.342 | |  | -2.1380 0.7431 |
| Income | 0.003 | | 0.087 | | 0.04 | 0.971 | |  | -0.1674 0.1737 |
| _cons | 11.312 | | 0.950 | | 11.91 | 0.000 | | *** | 9.4487 13.1743 |
|  | | | | | | | | |  |
| Mean dependent var | | 8.430 | | SD dependent var | | | 6.934 | |  |
| R-squared | | 0.091 | | Number of obs | | | 2085.000 | |  |
| F-test | | 18.808 | | Prob > F | | | 0.000 | |  |
| Akaike crit. (AIC) | | 13816.686 | | Bayesian crit. (BIC) | | | 13884.397 | |  |
|  | | | | | | | | |  |
| *** p<0.01, ** p<0.05, * p<0.1 | | | | | | | | |  |

**Table 12. Regression analysis of economic support and mental health over 80 years**

| Mentalhealth | Coef. | | St.Err | | t-value | p-value | Sig. | [95%conf.interval] |
| --- | --- | --- | --- | --- | --- | --- | --- | --- |
| Economic support | 0.368 | | 0.761 | | 0.48 | 0.629 |  | -1.1259 1.8618 |
| Gender | -0.668 | | 0.561 | | -1.19 | 0.234 |  | -1.7684 0.4323 |
| Spouse | 1.650 | | 0.546 | | 3.02 | 0.003 | *** | 0.5773 2.7218 |
| Education | 0.031 | | 0.361 | | 0.09 | 0.932 |  | -0.6771 0.7383 |
| Place of residence | 0.005 | | 0.653 | | 0.01 | 0.994 |  | -1.2776 1.2877 |
| Smoking | 0.871 | | 1.023 | | 0.85 | 0.395 |  | -1.1375 2.8788 |
| Drinking | 1.142 | | 0.584 | | 1.96 | 0.051 | * | -0.0041 2.2883 |
| SelfratedHealth | -0.359 | | 0.635 | | -0.56 | 0.572 |  | -1.6056 0.8877 |
| PensionInsurance | -0.679 | | 0.536 | | -1.27 | 0.206 |  | -1.7317 0.373 |
| HealthInsurance | -0.235 | | 0.983 | | -0.24 | 0.811 |  | -2.1635 1.6935 |
| Income | 0.159 | | 0.135 | | 1.18 | 0.240 |  | -0.1062 0.4235 |
| _cons | 5.362 | | 1.372 | | 3.91 | 0.000 | *** | 2.6687 8.0556 |
|  | | | | | | | |  |
| Mean dependent var | | 6.011 | | SD dependent var | | | 6.688 |  |
| R-squared | | 0.023 | | Number of obs | | | 856.000 |  |
| F-test | | 1.827 | | Prob > F | | | 0.046 |  |
| Akaike crit. (AIC) | | 5685.354 | | Bayesian crit. (BIC) | | | 5742.381 |  |
|  | | | | | | | |  |
| *** p<0.01, ** p<0.05, * p<0.1 | | | | | | | |  |

**Table 13. Regression results of female emotional support and 60-70 geriatric mental health**

| Mentalhealth | Coef. | | St.Err | | t-value | p-value | Sig. | | [95%conf.interval] |
| --- | --- | --- | --- | --- | --- | --- | --- | --- | --- |
| EmotionalSupport | -0.480 | | 0.219 | | -2.19 | 0.028 | ** | | -0.9101 -0.0507 |
| Gender | -1.308 | | 0.249 | | -5.25 | 0.000 | *** | | -1.7967 -0.8195 |
| Spouse | -0.019 | | 0.261 | | -0.07 | 0.942 |  | | -0.5309 0.4928 |
| Education | -0.816 | | 0.161 | | -5.08 | 0.000 | *** | | -1.1316 -0.5013 |
| Place of residence | -1.157 | | 0.300 | | -3.86 | 0.000 | *** | | -1.7451 -0.5693 |
| Smoking | 0.462 | | 0.552 | | 0.84 | 0.402 |  | | -0.6196 1.5441 |
| Drinking | -0.449 | | 0.249 | | -1.80 | 0.072 | * | | -0.9364 0.0393 |
| SelfratedHealth | -3.657 | | 0.258 | | -14.19 | 0.000 | *** | | -4.1627 -3.1519 |
| PensionInsurance | 1.125 | | 0.267 | | 4.22 | 0.000 | *** | | 0.6027 1.6481 |
| HealthInsurance | -0.508 | | 0.718 | | -0.71 | 0.479 |  | | -1.917 0.8999 |
| Income | 0.046 | | 0.062 | | 0.75 | 0.452 |  | | -0.0745 0.1673 |
| _cons | 12.183 | | 0.817 | | 14.91 | 0.000 | *** | | 10.5809 13.7851 |
|  | | | | | | | | |  |
| Mean dependent var | | 8.710 | | SD dependent var | | | | 6.822 |  |
| R-squared | | 0.115 | | Number of obs | | | | 3706.000 |  |
| F-test | | 43.778 | | Prob > F | | | | 0.000 |  |
| Akaike crit. (AIC) | | 24318.506 | | Bayesian crit. (BIC) | | | | 24393.119 |  |
|  | | | | | | | | |  |
| *** p<0.01, ** p<0.05, * p<0.1 | | | | | | | | |  |

**Table 14. Regression results of female emotional support and 70-80 geriatric mental health**

| Mentalhealth | Coef. | | St.Err | t-value | p-value | Sig. | [95%conf.interval] |
| --- | --- | --- | --- | --- | --- | --- | --- |
| EmotionalSupport | -1.136 | | 0.344 | -3.31 | 0.001 | *** | -1.8100 -0.4627 |
| Gender | -2.082 | | 0.348 | -5.98 | 0.000 | *** | -2.7654 -1.3988 |
| Spouse | -0.075 | | 0.321 | -0.23 | 0.815 |  | -0.7051 0.5547 |
| Education | -0.341 | | 0.226 | -1.51 | 0.132 |  | -0.7852 0.1029 |
| Place of residence | -0.967 | | 0.409 | -2.36 | 0.018 | ** | -1.7698 -0.1642 |
| Smoking | 0.280 | | 0.741 | 0.38 | 0.705 |  | -1.1739 1.7344 |
| Drinking | -0.743 | | 0.342 | -2.17 | 0.030 | ** | -1.4131 -0.0730 |
| SelfratedHealth | -3.372 | | 0.381 | -8.86 | 0.000 | *** | -4.1185 -2.6256 |
| PensionInsurance | 0.793 | | 0.345 | 2.29 | 0.022 | ** | 0.1150 1.4701 |
| HealthInsurance | -0.613 | | 0.732 | -0.84 | 0.402 |  | -2.0489 0.8222 |
| Income | -0.002 | | 0.087 | -0.02 | 0.985 |  | -0.1717 0.1685 |
| _cons | 12.185 | | 0.910 | 13.39 | 0.000 | *** | 10.401 13.9688 |
|  | | | | | | |  |
| Mean dependent var | | 8.430 | | SD dependent var | | 6.934 |  |
| R-squared | | 0.095 | | Number of obs | | 2085.000 |  |
| F-test | | 19.897 | | Prob > F | | 0.000 |  |
| Akaike crit. (AIC) | | 13805.764 | | Bayesian crit. (BIC) | | 13873.474 |  |
|  | | | | | | |  |
| *** p<0.01, ** p<0.05, * p<0.1 | | | | | | |  |

**Table 15. Regression results of female emotional support and mental health over 80 years**

| Mentalhealth | Coef. | | St.Err | t-value | p-value | | Sig. | [95%conf.interval] |
| --- | --- | --- | --- | --- | --- | --- | --- | --- |
| EmotionalSupport | 0.490 | | 0.595 | 0.82 | 0.411 | |  | -0.6787 1.6582 |
| Gender | -0.616 | | 0.566 | -1.09 | 0.277 | |  | -1.7268 0.4946 |
| Spouse | 1.644 | | 0.545 | 3.02 | 0.003 | | *** | 0.5754 2.7130 |
| Education | 0.001 | | 0.362 | 0.00 | 0.997 | |  | -0.7100 0.7129 |
| Place of residence | 0.013 | | 0.653 | 0.02 | 0.984 | |  | -1.2692 1.2959 |
| Smoking | 0.837 | | 1.024 | 0.82 | 0.414 | |  | -1.1718 2.8462 |
| Drinking | 1.154 | | 0.584 | 1.98 | 0.048 | | ** | 0.0081 2.3004 |
| SelfratedHealth | -0.371 | | 0.634 | -0.58 | 0.559 | |  | -1.616 0.8743 |
| PensionInsurance | -0.646 | | 0.537 | -1.20 | 0.229 | |  | -1.6986 0.4075 |
| HealthInsurance | -0.282 | | 0.982 | -0.29 | 0.774 | |  | -2.2090 1.6451 |
| Income | 0.156 | | 0.135 | 1.16 | 0.247 | |  | -0.1086 0.4212 |
| _cons | 5.350 | | 1.250 | 4.28 | 0.000 | | *** | 2.8965 7.8043 |
|  | | | | | | | |  |
| Mean dependent var | | 6.011 | | SD dependent var | | 6.688 | |  |
| R-squared | | 0.024 | | Number of obs | | 856.000 | |  |
| F-test | | 1.869 | | Prob > F | | 0.040 | |  |
| Akaike crit. (AIC) | | 5684.905 | | Bayesian crit. (BIC) | | 5741.932 | |  |
|  | | | | | | | |  |
| *** p<0.01, ** p<0.05, * p<0.1 | | | | | | | |  |

**Table 16. Regression results of caring support and 60-70 geriatric mental health**

| Mentalhealth | Coef. | | St.Err | t-value | | p-value | Sig. | [95%conf.interval] |
| --- | --- | --- | --- | --- | --- | --- | --- | --- |
| CaringSuppor | -0.295 | | 0.212 | -1.39 | | 0.164 |  | -0.7119 0.1210 |
| Gender | -1.302 | | 0.249 | -5.22 | | 0.000 | *** | -1.7912 -0.8134 |
| Spouse | -0.054 | | 0.261 | -0.21 | | 0.837 |  | -0.5650 0.4574 |
| Education | -0.824 | | 0.161 | -5.13 | | 0.000 | *** | -1.1399 -0.5090 |
| Place of residence | -1.139 | | 0.300 | -3.79 | | 0.000 | *** | -1.7275 -0.5503 |
| Smoking | 0.462 | | 0.552 | 0.84 | | 0.402 |  | -0.6170 1.5447 |
| Drinking | -0.445 | | 0.249 | -1.79 | | 0.074 | * | -0.9330 0.0431 |
| SelfratedHealth | -3.657 | | 0.258 | -14.18 | | 0.000 | *** | -4.1622 -3.1509 |
| PensionInsurance | 1.152 | | 0.267 | 4.32 | | 0.000 | *** | 0.6288 1.6758 |
| HealthInsurance | -0.533 | | 0.719 | -0.74 | | 0.458 |  | -1.9425 0.8756 |
| Income | 0.043 | | 0.062 | 0.69 | | 0.487 |  | -0.0780 0.1638 |
| _cons | 12.076 | | 0.817 | 14.78 | | 0.000 | *** | 10.4741 13.6774 |
|  | | | | | | | |  |
| Mean dependent var | | 8.710 | | | SD dependent var | | 6.822 |  |
| R-squared | | 0.115 | | | Number of obs | | 3706.000 |  |
| F-test | | 43.484 | | | Prob > F | | 0.000 |  |
| Akaike crit. (AIC) | | 24321.382 | | | Bayesian crit. (BIC) | | 24395.994 |  |
|  | | | | | | | |  |
| *** p<0.01, ** p<0.05, * p<0.1 | | | | | | | |  |

**Table 17. Regression results of caring support and 70-80 geriatric mental health**

| Mentalhealth | Coef. | | St.Err | t-value | p-value | Sig. | [95%conf.interval] |
| --- | --- | --- | --- | --- | --- | --- | --- |
| CaringSuppor | -0.535 | | 0.296 | -1.81 | 0.071 | * | -1.1156 0.0450 |
| Gender | -1.975 | | 0.347 | -5.69 | 0.000 | *** | -2.6563 -1.2941 |
| Spouse | -0.158 | | 0.322 | -0.49 | 0.623 |  | -0.7895 0.4730 |
| Education | -0.395 | | 0.227 | -1.74 | 0.082 | * | -0.8403 0.0496 |
| Place of residence | -0.974 | | 0.410 | -2.37 | 0.018 | ** | -1.7781 -0.1695 |
| Smoking | 0.282 | | 0.743 | 0.38 | 0.704 |  | -1.1747 1.7390 |
| Drinking | -0.743 | | 0.343 | -2.17 | 0.030 | ** | -1.4147 -0.0712 |
| SelfratedHealth | -3.399 | | 0.382 | -8.91 | 0.000 | *** | -4.1474 -2.6508 |
| PensionInsurance | 0.825 | | 0.346 | 2.38 | 0.017 | ** | 0.1463 1.5036 |
| HealthInsurance | -0.736 | | 0.733 | -1.00 | 0.316 |  | -2.1749 0.7021 |
| Income | 0.006 | | 0.087 | 0.07 | 0.942 |  | -0.1641 0.1767 |
| _cons | 11.805 | | 0.908 | 13.00 | 0.000 | *** | 10.0238 13.5853 |
|  | | | | | | |  |
| Mean dependent var | | 8.430 | | SD dependent var | | 6.934 |  |
| R-squared | | 0.092 | | Number of obs | | 2085.000 |  |
| F-test | | 19.130 | | Prob > F | | 0.000 |  |
| Akaike crit. (AIC) | | 13813.452 | | Bayesian crit. (BIC) | | 13881.163 |  |
|  | | | | | | |  |
| *** p<0.01, ** p<0.05, * p<0.1 | | | | | | |  |

**Table 18. Regression results of caring support and geriatric mental health over 80 years**

| Mentalhealth | Coef. | | St.Err | t-value | | p-value | Sig. | [95%conf.interval] |
| --- | --- | --- | --- | --- | --- | --- | --- | --- |
| CaringSuppor | -0.720 | | 0.482 | -1.50 | | 0.135 |  | -1.6661 0.2251 |
| Gender | -0.666 | | 0.558 | -1.20 | | 0.232 |  | -1.7609 0.4281 |
| Spouse | 1.603 | | 0.545 | 2.94 | | 0.003 | *** | 0.5335 2.6721 |
| Education | -0.002 | | 0.361 | -0.01 | | 0.996 |  | -0.7101 0.7064 |
| Place of residence | -0.051 | | 0.654 | -0.08 | | 0.937 |  | -1.3340 1.2315 |
| Smoking | 0.926 | | 1.023 | 0.91 | | 0.365 |  | -1.0812 2.9332 |
| Drinking | 1.102 | | 0.584 | 1.89 | | 0.059 | * | -0.0440 2.2482 |
| SelfratedHealth | -0.355 | | 0.634 | -0.56 | | 0.576 |  | -1.5989 0.8899 |
| PensionInsurance | -0.654 | | 0.535 | -1.22 | | 0.222 |  | -1.70499 0.3964 |
| HealthInsurance | -0.227 | | 0.981 | -0.23 | | 0.817 |  | -2.1513 1.6981 |
| Income | 0.161 | | 0.135 | 1.20 | | 0.232 |  | -0.1035 0.4256 |
| _cons | 6.211 | | 1.220 | 5.09 | | 0.000 | *** | 3.8174 8.6054 |
|  | | | | | | | |  |
| Mean dependent var | | 6.011 | | | SD dependent var | | 6.688 |  |
| R-squared | | 0.026 | | | Number of obs | | 856.000 |  |
| F-test | | 2.014 | | | Prob > F | | 0.025 |  |
| Akaike crit. (AIC) | | 5683.326 | | | Bayesian crit. (BIC) | | 5740.353 |  |
|  | | | | | | | |  |
| *** p<0.01, ** p<0.05, * p<0.1 | | | | | | | |  |

**Table 19. Regression analysis of economic support and rural mental health**

| Mentalhealth | Coef. | | St.Err | t-value | | p-value | Sig. | [95%conf.interval] |
| --- | --- | --- | --- | --- | --- | --- | --- | --- |
| Economic support | 0.066 | | 0.282 | 0.23 | | 0.815 |  | -0.4867 0.6184 |
| Age | -1.554 | | 0.147 | -10.54 | | 0.000 | *** | -1.8427 -1.2645 |
| Gender | -1.595 | | 0.227 | -7.01 | | 0.000 | *** | -2.0405 -1.1488 |
| Spouse | 0.148 | | 0.223 | 0.66 | | 0.507 |  | -0.2891 0.5852 |
| Education | -0.512 | | 0.152 | -3.36 | | 0.001 | *** | -0.8105 -0.2129 |
| Smoking | -0.045 | | 0.513 | -0.09 | | 0.931 |  | -1.0511 0.9620 |
| Drinking | -0.260 | | 0.225 | -1.16 | | 0.247 |  | -0.7009 0.1804 |
| SelfratedHealth | -3.122 | | 0.246 | -12.68 | | 0.000 | *** | -3.6049 -2.6392 |
| PensionInsurance | 0.593 | | 0.236 | 2.51 | | 0.012 | ** | 0.1301 1.0557 |
| HealthInsurance | -0.531 | | 0.497 | -1.07 | | 0.286 |  | -1.5049 0.4437 |
| Income | 0.063 | | 0.061 | 1.04 | | 0.298 |  | -0.0557 0.1820 |
| _cons | 13.273 | | 0.697 | 19.03 | | 0.000 | *** | 11.9057 14.6399 |
|  | | | | | | | |  |
| Mean dependent var | | 8.741 | | | SD dependent var | | 6.999 |  |
| R-squared | | 0.075 | | | Number of obs | | 5034.000 |  |
| F-test | | 37.257 | | | Prob > F | | 0.000 |  |
| Akaike crit. (AIC) | | 33503.858 | | | Bayesian crit. (BIC) | | 33582.146 |  |
|  | | | | | | | |  |
| *** p<0.01, ** p<0.05, * p<0.1 | | | | | | | |  |

**Table 20. Regression analysis of economic support and urban elderly people**

| Mentalhealth | Coef. | | St.Err | | t-value | p-value | | Sig. | [95%conf.interval] |
| --- | --- | --- | --- | --- | --- | --- | --- | --- | --- |
| Economic support | 0.050 | | 0.359 | | 0.14 | 0.889 | |  | -0.6539 0.7543 |
| Age | -0.406 | | 0.233 | | -1.74 | 0.083 | | * | -0.8635 0.0524 |
| Gender | -0.873 | | 0.360 | | -2.43 | 0.015 | | ** | -1.5789 -0.1676 |
| Spouse | 0.285 | | 0.360 | | 0.79 | 0.429 | |  | -0.4218 0.9919 |
| Education | -0.400 | | 0.213 | | -1.87 | 0.061 | | * | -0.8187 0.0187 |
| Smoking | 1.348 | | 0.657 | | 2.05 | 0.040 | | ** | 0.0600 2.6357 |
| Drinking | -0.810 | | 0.359 | | -2.25 | 0.024 | | ** | -1.5142 -0.1054 |
| SelfratedHealth | -3.636 | | 0.357 | | -10.19 | 0.000 | | *** | -4.3353 -2.9359 |
| PensionInsurance | 1.144 | | 0.365 | | 3.13 | 0.002 | | *** | 0.4272 1.8601 |
| HealthInsurance | -1.624 | | 1.224 | | -1.33 | 0.185 | |  | -4.0249 0.7765 |
| Income | 0.013 | | 0.074 | | 0.18 | 0.856 | |  | -0.1316 0.15842 |
| _cons | 10.936 | | 1.445 | | 7.57 | 0.000 | | *** | 8.1015 13.7699 |
|  | | | | | | | | |  |
| Mean dependent var | | 6.818 | | SD dependent var | | | 6.349 | |  |
| R-squared | | 0.097 | | Number of obs | | | 1613.000 | |  |
| F-test | | 15.634 | | Prob > F | | | 0.000 | |  |
| Akaike crit. (AIC) | | 10398.342 | | Bayesian crit. (BIC) | | | 10462.972 | |  |
|  | | | | | | | | |  |
| *** p<0.01, ** p<0.05, * p<0.1 | | | | | | | | |  |

**Table 21. Regression analysis of emotional support and rural mental health**

| Mentalhealth | Coef. | | St.Err | t-value | | p-value | Sig. | [95%conf.interval] |
| --- | --- | --- | --- | --- | --- | --- | --- | --- |
| EmotionalSupport | -0.570 | | 0.209 | -2.73 | | 0.006 | *** | -0.9790 -0.1609 |
| Age | -1.486 | | 0.148 | -10.02 | | 0.000 | *** | -1.7760 -1.1950 |
| Gender | -1.654 | | 0.227 | -7.29 | | 0.000 | *** | -2.0987 -1.2092 |
| Spouse | 0.189 | | 0.222 | 0.85 | | 0.396 |  | -0.2470 0.6246 |
| Education | -0.490 | | 0.152 | -3.22 | | 0.001 | *** | -0.7893 -0.1917 |
| Smoking | -0.026 | | 0.513 | -0.05 | | 0.959 |  | -1.0323 0.9794 |
| Drinking | -0.276 | | 0.225 | -1.23 | | 0.219 |  | -0.7162 0.1644 |
| SelfratedHealth | -3.122 | | 0.246 | -12.68 | | 0.000 | *** | -3.6042 -2.6392 |
| PensionInsurance | 0.575 | | 0.236 | 2.44 | | 0.015 | ** | 0.1131 1.0372 |
| HealthInsurance | -0.501 | | 0.496 | -1.01 | | 0.313 |  | -1.4742 0.4717 |
| Income | 0.063 | | 0.061 | 1.04 | | 0.297 |  | -0.0556 0.1819 |
| _cons | 13.570 | | 0.681 | 19.93 | | 0.000 | *** | 12.2351 14.9045 |
|  | | | | | | | |  |
| Mean dependent var | | 8.741 | | | SD dependent var | | 6.999 |  |
| R-squared | | 0.077 | | | Number of obs | | 5034.000 |  |
| F-test | | 37.985 | | | Prob > F | | 0.000 |  |
| Akaike crit. (AIC) | | 33496.440 | | | Bayesian crit. (BIC) | | 33574.727 |  |
|  | | | | | | | |  |
| *** p<0.01, ** p<0.05, * p<0.1 | | | | | | | |  |

**Table 22. Regression analysis of emotional support and urban mental health**

| Mentalhealth | Coef. | | St.Err | t-value | | p-value | Sig. | [95%conf.interval] |
| --- | --- | --- | --- | --- | --- | --- | --- | --- |
| EmotionalSupport | -0.322 | | 0.340 | -0.95 | | 0.344 |  | -0.9898 0.3457 |
| Age | -0.359 | | 0.235 | -1.53 | | 0.127 |  | -0.8193 0.1021 |
| Gender | -0.883 | | 0.360 | -2.46 | | 0.014 | ** | -1.5888 -0.1775 |
| Spouse | 0.298 | | 0.360 | 0.83 | | 0.407 |  | -0.4080 1.0049 |
| Education | -0.405 | | 0.213 | -1.90 | | 0.058 | * | -0.8226 0.0134 |
| Smoking | 1.323 | | 0.657 | 2.01 | | 0.044 | ** | 0.0345 2.6115 |
| Drinking | -0.806 | | 0.359 | -2.25 | | 0.025 | ** | -1.5103 -0.1019 |
| SelfratedHealth | -3.632 | | 0.357 | -10.19 | | 0.000 | *** | -4.3314 -2.9327 |
| PensionInsurance | 1.148 | | 0.364 | 3.15 | | 0.002 | *** | 0.4332 1.8624 |
| HealthInsurance | -1.611 | | 1.224 | -1.32 | | 0.188 |  | -4.0113 0.7888 |
| Income | 0.015 | | 0.074 | 0.21 | | 0.834 |  | -0.1295 0.1605 |
| _cons | 11.118 | | 1.438 | 7.73 | | 0.000 | *** | 8.2980 13.9379 |
|  | | | | | | | |  |
| Mean dependent var | | 6.818 | | | SD dependent var | | 6.349 |  |
| R-squared | | 0.097 | | | Number of obs | | 1613.000 |  |
| F-test | | 15.722 | | | Prob > F | | 0.000 |  |
| Akaike crit. (AIC) | | 10397.460 | | | Bayesian crit. (BIC) | | 10462.091 |  |
|  | | | | | | | |  |
| *** p<0.01, ** p<0.05, * p<0.1 | | | | | | | |  |

**Table 23. Regression analysis of caring support and rural mental health**

| Mentalhealth | Coef. | | St.Err | t-value | p-value | Sig. | [95%conf.interval] |
| --- | --- | --- | --- | --- | --- | --- | --- |
| CaringSuppor | -0.408 | | 0.193 | -2.12 | 0.034 | ** | -0.7863 -0.0303 |
| Age | -1.518 | | 0.147 | -10.32 | 0.000 | *** | -1.8065 -1.230 |
| Gender | -1.611 | | 0.226 | -7.13 | 0.000 | *** | -2.0547 -1.1681 |
| Spouse | 0.141 | | 0.222 | 0.64 | 0.525 |  | -0.2940 0.5765 |
| Education | -0.520 | | 0.152 | -3.42 | 0.001 | *** | -0.8191 -0.2218 |
| Smoking | -0.031 | | 0.513 | -0.06 | 0.952 |  | -1.0373 0.9749 |
| Drinking | -0.276 | | 0.225 | -1.23 | 0.219 |  | -0.7170 0.1642 |
| SelfratedHealth | -3.127 | | 0.246 | -12.70 | 0.000 | *** | -3.6095 -2.6442 |
| PensionInsurance | 0.605 | | 0.236 | 2.57 | 0.010 | ** | 0.1427 1.0667 |
| HealthInsurance | -0.538 | | 0.496 | -1.08 | 0.279 |  | -1.5110 0.4352 |
| Income | 0.062 | | 0.061 | 1.01 | 0.310 |  | -0.0573 0.1803 |
| _cons | 13.530 | | 0.682 | 19.83 | 0.000 | *** | 12.1928 14.8677 |
|  | | | | | | |  |
| Mean dependent var | | 8.741 | | SD dependent var | | 6.999 |  |
| R-squared | | 0.076 | | Number of obs | | 5034.000 |  |
| F-test | | 37.692 | | Prob > F | | 0.000 |  |
| Akaike crit. (AIC) | | 33499.421 | | Bayesian crit. (BIC) | | 33577.708 |  |
|  | | | | | | |  |
| *** p<0.01, ** p<0.05, * p<0.1 | | | | | | |  |

**Table 24. Regression analysis of caring support and urban mental health**

| Mentalhealth | Coef. | | St.Err | | t-value | p-value | | Sig. | [95%conf.interval] |
| --- | --- | --- | --- | --- | --- | --- | --- | --- | --- |
| CaringSuppor | -0.410 | | 0.307 | | -1.34 | 0.181 | |  | -1.0113 0.1911 |
| Age | -0.393 | | 0.231 | | -1.70 | 0.089 | | * | -0.8452 0.0597 |
| Gender | -0.896 | | 0.360 | | -2.49 | 0.013 | | ** | -1.6016 -0.1898 |
| Spouse | 0.263 | | 0.360 | | 0.73 | 0.466 | |  | -0.4440 0.9695 |
| Education | -0.417 | | 0.213 | | -1.95 | 0.051 | | * | -0.8351 0.0018 |
| Smoking | 1.338 | | 0.656 | | 2.04 | 0.042 | | ** | 0.0509 2.6253 |
| Drinking | -0.816 | | 0.359 | | -2.27 | 0.023 | | ** | -1.5205 -0.1125 |
| SelfratedHealth | -3.629 | | 0.356 | | -10.18 | 0.000 | | *** | -4.3281 -2.9298 |
| PensionInsurance | 1.182 | | 0.365 | | 3.24 | 0.001 | | *** | 0.4657 1.8981 |
| HealthInsurance | -1.707 | | 1.225 | | -1.39 | 0.164 | |  | -4.1090 0.6958 |
| Income | 0.016 | | 0.074 | | 0.22 | 0.827 | |  | -0.1288 0.1612 |
| _cons | 11.301 | | 1.450 | | 7.79 | 0.000 | | *** | 8.4569 14.1456 |
|  | | | | | | | | |  |
| Mean dependent var | | 6.818 | | SD dependent var | | | 6.349 | |  |
| R-squared | | 0.098 | | Number of obs | | | 1613.000 | |  |
| F-test | | 15.812 | | Prob > F | | | 0.000 | |  |
| Akaike crit. (AIC) | | 10396.559 | | Bayesian crit. (BIC) | | | 10461.189 | |  |
|  | | | | | | | | |  |
| *** p<0.01, ** p<0.05, * p<0.1 | | | | | | | | |  |
